# Agonists and hydrogen peroxide mediate hyperoxidation of β2-adrenergic receptor in airway epithelial cells: Implications for tachyphylaxis to β2-agonists in constrictive airway disorders

**DOI:** 10.1016/j.biopha.2023.115763

**Published:** 2023-10-20

**Authors:** Kirti Singh, Razan L. Teyani, Nader H. Moniri

**Affiliations:** a Department of Pharmaceutical Sciences, College of Pharmacy, Mercer University Health Sciences Center, Mercer University, Atlanta, GA 30341, USA; b Department of Biomedical Sciences, School of Medicine, Mercer University Health Sciences Center, Mercer University, Macon, GA 31207, USA

**Keywords:** Beta-adrenergic receptors, Reactive oxygen species, Asthma, Airway epithelial cells, Cyclic AMP

## Abstract

Asthma and other airway obstructive disorders are characterized by heightened inflammation and excessive airway epithelial cell reactive oxygen species (ROS), which give rise to a highly oxidative environment. After decades of use, β2-adrenergic receptor (β2AR) agonists remain at the forefront of treatment options for asthma, however, chronic use of β2-agonists leads to tachyphylaxis to the bronchorelaxant effects, a phenomenon that remains mechanistically unexplained. We have previously demonstrated that β2AR agonism increases ROS generation in airway epithelial cells, which upholds proper receptor function via feedback oxidation of β2AR cysteine thiolates to Cys-S-sulfenic acids (Cys-SOH). Our previous results also demonstrate that prevention of normal redox cycling of this post-translational oxi-modification back to the thiol prevents proper receptor function. Given that Cys-S-sulfenic acids can be irreversibly overoxidized to Cys-S-sulfinic (Cys-SO_2_H) or S-sulfonic (Cys-SO_3_H) acids, which are incapable of further participation in redox reactions, we hypothesized that β2-agonist tachyphylaxis may be explained by hyperoxidation of β2AR to S-sulfinic acids. Here, using airway epithelial cell lines and primary small airway epithelial cells from healthy and asthma-diseased donors, we show that β2AR agonism generates H_2_O_2_ in a receptor and NAPDH oxidase-dependent manner. We also demonstrate that acute and chronic receptor agonism can facilitate β2AR S-sulfination, and that millimolar H_2_O_2_ concentrations are deleterious to β2AR-mediated cAMP formation, an effect that can be rescued to a degree in the presence of the cysteine-donating antioxidant *N*-acetyl-_*L*_-cysteine. Our results reveal that the oxidative state of β2AR may contribute to receptor functionality and may, at least in part, explain β2-agonist tachyphylaxis.

## 1. Introduction

Inhaled β2-adrenergic receptor (β2AR) agonists remain the gold-standard for treatment of bronchoconstrictive pulmonary disorders such as asthma or chronic obstructive pulmonary disease (COPD), which are characterized by hypercontractility of smooth muscles that constrict airways leading to dyspnea, chest tightening, coughing and wheezing. Agonism of β2AR facilitates relaxation of the airways and a variety of β2AR agonists including salbutamol (albuterol), formoterol, and salmeterol are used clinically for the treatment of these disorders. However, an abundance of literature demonstrates that chronic use of β2-agonists leads to tachyphylaxis (i.e., tolerance) to the bronchorelaxant response, an effect that has been proposed to contribute to morbidity and mortality [1–3]. Although a variety of hypotheses have been proposed to explain the phenomenon of β2-agonist tachyphylaxis, including β2AR polymorphisms, or desensitization and internalization downstream of chronic agonism, none have been shown to fully account for this effect and the exact mechanisms remain elusive [1–3].

While smooth muscle contraction causes increased airway resistance and subsequent symptomology of obstructive airway disorders, it is now well-accepted that the etiology of asthma is driven at least in part due to airway epithelial cell dysfunction that facilitates heightened inflammation and oxidative stress, which greatly influences the underlying smooth muscle [4–9]. While both airway epithelial and smooth muscle cells express β2AR, historically, the smooth muscle, rather than epithelial cells, have been the subject of extensive study as far as the receptor is concerned. However, recent evidence suggests that oxidative stress, particularly elevated levels of superoxide and hydrogen peroxide (H_2_O_2_) specifically derived from the airway epithelium, play a significant role towards airway inflammation and smooth muscle contraction that yields the asthmatic phenotype [5,10,11]. Since the airway epithelium is in direct contact with, and responds swiftly to inhaled substances, inhaled environmental sources and triggers of reactive oxygen species (ROS) production, such as pollution and allergens, can contribute to epithelial ROS generation. In addition, infiltration of immune cells, chiefly neutrophils and eosinophils, which localize to the airway, also contribute to significant ROS burdens [5,10,11]. Importantly, the membrane bound NADPH oxidase (NOX) family of enzymes are a significant source of ROS generation in the airway epithelium, and upregulation of the NOX4 isoform in particular is thought to play a major role in over-production of ROS in obstructive airway disorders [12,13]. NOX4 is also unique in that it is constitutively active in the absence of upstream activators and can also directly produce H_2_O_2_, whereas the other NOX isoforms only generate superoxide, which must then be enzymatically converted to H_2_O_2_ by superoxide dismutase (SOD) [14–16].

We have previously shown that agonism of β2AR generates intracellular ROS in a NOX-dependent manner in clonal cell lines as well as in human lung epithelial cells [17–20], and these results have been verified by others in a variety of cell types and tissues [21–27]. Our previous work also demonstrates that some level of ROS are required for proper β2AR function, as ROS sequestration or NOX inhibition decreases both G-protein and β-arrestin-dependent β2AR signaling [19,20]. Recently, we have also shown that β2AR-mediated ROS generation, as well as exposure to exogenous H_2_O_2_ can oxidize β2AR cysteine residues forming transient cysteine-S-sulfenic acids (-Cys-SOH), a first-order oxidative post-translational modification [18,28]. Importantly, in the presence of high concentrations or prolonged exposure to ROS, S-sulfenic acids can be further oxidized to form higher-order cysteine S-sulf*i*nic (Cys-SO_2_H) or S-sulf*i*nic (Cys-SO_3_H) acids, which are stable and generally irreversible, and can lead to protein dysfunction as seen in oxidative stress [29–32]. Consistent with this, our previous work revealed that while cysteine-S-sulfenic acid oxidized β2AR exhibits enhanced ligand binding and improved downstream function, irreversible “trapping” of the β2AR-cysteine-S-sulfenic acid with the selective Cys-SOH alkylator dimedone, which mimics S-sulfination/S-sulfonation and prevents normal redox recycling back to the native thiol state, inhibits ligand binding and decreases downstream β2AR signaling [18]. These results demonstrated that the ROS-sensitive cysteine residue(s) in β2AR regulate its function, and suggest that overoxidation of these residues from Cys-S-sulfenic to Cys-S-sulfinic acids may lead to receptor dysfunction. Given this effect along with the known ability of β2AR to generate ROS upon its agonism, and the well-described elevation in ROS in asthma, we hypothesize that ROS can post-translationally oxidize β2AR to Cys-S-sulfinic acids. Furthermore, we hypothesize that chronic agonism and heightened ROS in asthma could potentially contribute to β2-agonist tachyphylaxis via this irreversible oxomodification of β2AR, which inhibits its function. In this study, we have assessed β2AR-induced H_2_O_2_ generation in human airway epithelial cell lines as well as in primary healthy and asthma-diseased human small airway epithelial cells (SAEC) and for the first time, we reveal β2AR agonist and H_2_O_2_-induced S-sulfination of β2AR, suggesting that this over-oxidation of β2AR may contribute to lack of β2AR function seen in β2-agonist tachyphylaxis. Our results also denote interesting differences in healthy versus asthma-diseased SAEC, most notably, significant upregulation of the cAMP-metabolizing enzyme PDE4.

## 2. Materials and methods

### 2.1. Chemicals and reagents

Amplex Red and AbGreen-indicator H_2_O_2_ detection reagents were purchased from Thermo Fisher Scientific (A12222) (Waltham, MA) and Abcam (ab138874) (Waltham, MA), respectively. DiaAlk (PubChem CID: 134688955) (1-(tert-Butyl) 2-(2-methyl-4-(prop-2-yn-1-yloxy) butan-2-yl) (*E*)-diazene-1,2-dicarboxylate, 8 mM in DMSO) was acquired from Aobious Inc (Gloucester, MA), biotin-azide (3aS,4 S,6aR)-N-(3-azidopropyl) hexahydro-2-oxo-1 H-thieno[3,4-*d*] imidazole-4-pentanamide, 5 mM in DMSO) and VAS2870 (PubChem CID: 4058452) were purchased from Cayman Chemicals (Ann Arbor, MI), (−)-isoproterenol bitartrate (ISO, PubChem CID:160420) (I2760), salbutamol (SAL, PubChem CID: 2083) (S8260), and *N*-Acetyl-_*L*_-cysteine (NAC, PubChem CID:12035) (A7250) were purchased from Millipore Sigma (St. Louis, MO); 3-isobutyl-1-methylxanthanine (IBMX, PubChem CID:3758) (PHZ1124), forskolin (FSK, PubChem CID:47936) (BP252010), dithiothreitol (DTT, PubChem CID:446094), and 4,4′-dithiodipyridine (4,4-DPS, PubChem CID: 75846) (162240050) were purchased from Thermo Fisher (Carlsbad, CA). ICI-118,551 hydrochloride (PubChem CID: 11957590) (0821) was from Tocris Bioscience (Bristol, UK). All other reagents were purchased at their highest available purity from Millipore Sigma or Fisher Scientific.

### 2.2. Cell culture and patient characteristics

Primary human small airway epithelial cells from asthma-diseased (A-SAEC) and healthy (SAEC) patient donors were obtained from Lonza (CC-2547/2932, Basel, Switzerland) and cultured in small airway cell basal medium (SABM) (CC-3119) supplemented with bovine pituitary extract 2.0 ml, insulin 0.5 ml, hydrocortisone 0.5 ml, gentamicin sulfate 0.5 ml, retinoic acid 0.5 ml, fatty acid-free bovine serum albumin (BSA) 5 ml, transferrin 0.5 ml, triiodothyronine 0.5 ml, epinephrine 0.5 ml, and human epidermal growth factor 0.5 ml, as supplied by the manufacturer, and grown in a humidified atmosphere at 37 °C in 5% CO_2_. To minimize variability between patients and contamination of other cell types, we utilized commercially available primary epithelial cells from donors, which are commercially validated to contain greater than 90% small airway epithelial cells as assessed by cytokeratin-19 expression. The same patient-derived reserved lots were used throughout the experiments to ensure consistency. The healthy SAEC were obtained from a 25-year-old Caucasian female (100.7 kg, 63” height) while the asthma-diseased SAEC were obtained from a 15-year-old Caucasian female (66 kg, 68” height), both of whom were non-diabetic, with no history of cardiovascular disease, hypertension, or smoking or alcohol use. The asthmatic patient was diagnosed at age two, was on albuterol twice per month, and deceased at age 15 due to a respiratory issue that was unspecified due to confidentiality. Cells were passaged a maximum of 6–7 times, following which, another commercially-acquired cryopreserved parenteral aliquot of the same lot reserve was used. Human lung airway epithelial cells (CALU3 and A549) were obtained from ATCC (Manassas, VA), cultured in Dulbecco’s modified Eagles medium (DMEM) supplemented with 10% fetal bovine serum and 1% penicillin-streptomycin (Life Technologies, Grand Island, NY) and F-12 K media supplemented with 10% fetal bovine serum, respectively.

### 2.3. Real-time cyclic AMP formation

Cells were transiently transfected with 8 μg of GloSensor-22 F cAMP plasmid (Promega, Madison, WI) using TurboFectin 8.0 (Origene, Rockville, MD) for 24 h, following the manufacturer’s instructions. Twenty-four hours following transfection, cells were trypsinized, resuspended in DMEM with 25 mM HEPES and 10% FBS and centrifuged at 250 × *g*, for 5 min at 4 °C. The cell pellet was resuspended in media above supplemented with 2% GloSensor reagent at 3 × 10^5^ cells/ml, and incubated for 2 h at room temperature in dark with gentle agitation every 15 min to avoid settling. Cells were loaded at 3 × 10^4^ cells/well in white 96-well plates and pretreated with 100 μM IBMX for 5 min prior to agonism with ISO, or other agents, as noted in the figure legends. Where ICI-118,551 was assessed, cells were treated for 5 min prior or 10 min following ISO addition, as indicated in the figure legends. Of note, since this is a real-time measurement, higher concentrations of agents were used than what is required in endpoint measurements, given the continuous presence of ISO throughout the course of study. Where the role of oxidants was assessed, cells were treated with indicated concentrations of H_2_O_2_ for 1 min prior or 10 min after ISO stimulation, as described in the figure legends. Luminescence was measured using MicroBeta2 2450 Microplate counter (Perkin Elmer, Waltham, MA) and cell viability was determined using automated cell counting in the presence of 0.4% trypan blue, 4 h following agonism with isoproterenol. In initial studies, treatment with 0.1–1 mM concentrations of H_2_O_2_ for 30–180 min had no deleterious effects on cell viability as detected by cell viability assays (supplementary figure 1). In some experiments, to ensure functional viability, cells were restimulated with IBMX (100 μM) and FSK (10 μM) 4 h following agonism with ISO.

### 2.4. Real-time H_2_O_2_ generation

Confluent SAEC, CALU3 and A549 cells were trypsinized, resuspended in serum-containing media and centrifuged at 250 × *g* at 4 °C for 5 min. Cells were resuspended in assay buffer (140 mM NaCl, 2.7 mM KCl, 1 mM MgCl_2_, 1 mM CaCl_2_, 0.37 mM NaH_2_PO_4_, 24 mM NaHCO_3_, 25 mM HEPES, 0.1% Glucose, pH 7.4) at 3.5 × 10^4^ cells in 100 μl in a black 96-well plate prior to the addition of the cell-impermeable, extracellular H_2_O_2_-probe Amplex Red and stimulation with ISO or H_2_O_2_, as indicated in figure legends. Of note, since this is a real-time measurement, higher concentrations of agents were used than what is required in endpoint measurements, given the continuous presence of ISO throughout the course of study. Where VAS2870 was used, a final concentration of 5 μM was added prior to Amplex Red. Exogenously applied H_2_O_2_ (0.1 μM) was utilized as an internal positive control in all the experiments and detection of fluorescence, a marker of H_2_O_2_ generation was measured at excitation/emission wavelength of 545 nm/590 nm using Tecan M200 Infinite Pro plate reader (Tecan, Baldwin Park, CA) for 60 min. The generation of intracellular H_2_O_2_ was also assessed using the cell-permeable, H_2_O_2_-specific fluorescent AbGreen indicator (Abcam). Here, 3 × 10^4^ cells were seeded in 96-well black clear bottom plates in 100 μl media, and 24 h later, cells were washed three times with PBS and incubated with the probe for 60 min, following the manufacturer’s instructions. Cells were treated with ISO as indicated and images were acquired and quantification and statistical analysis was performed on the representative 4X images using the respective monochrome fluorescent images to determine the average fluorescence intensity of the selected area (2.0–2.1 mm^2^) using an ECHO Revolve fluorescent microscope (Discover Echo, San Diego, CA). Where inhibitors were used, they were preincubated for 30 min prior to ISO addition, and 0.1 μM H_2_O_2_ and water were used as positive and negative controls, respectively.

### 2.5. Detection β_2_AR cysteine-S-sulfinic acids

To examine the agonist and H_2_O_2_ mediated formation of cysteine-S-sulfinic acids, SAEC were assessed in both acute (single treatment up to 60 min) or chronic (twice daily treatment for seven days) treatment paradigms, as indicated in the figure legends. Following treatments, cells were washed with iced PBS, lysed in modified RIPA buffer (50 mM Triethanolamine, 150 mM NaCl, 1% NP-40, 1% sodium deoxycholate, 0.5% SDS, 200 U/ml catalase, pH 7.4) and 1X EDTA-free HALT protease inhibitor for 20 min with gentle agitation. After clearing by centrifugation, the lysate was incubated with 40 μl neutravidin beads for one hour at room temperature with constant tumbling to preclear endogenously biotinylated proteins in the sample. After standardization of the protein concentrations to 5 mg/ml, 20 μl was removed for detection of β_2_AR and β-actin in the reaction input, and 50 μl was tumbled with 200 mM 4,4-DPS for 1 h at room temperature to block free-thiols. Samples were cleared using Bio-Spin P-30 gel columns (BioRad Laboratories, Hercules, CA), pre-equilibrated with 100 mM HEPES and 100 mM NaCl, pH 8.5. Lysates were tumbled with the clickable selective cysteine-S-sulfinic acid probe DiaAlk (1 mM) with 0.5% SDS for 2 h at room temperature in dark, and subsequently incubated with 1 mM DTT for 30 min to quench the reaction, then passed through detergent-removal column (Pierce) pre-equilibrated with 100 mM PBS, pH 7.4. Buffer exchanged samples were subjected to click chemistry as we have described previously [17], and below. After biotinylating DiaAlk labeled residues using click chemistry, lysates were then subjected to chloroform-methanol precipitation, air dried, and redissolved in 1 ml dilution RIPA buffer (25 mM Tris-HCl, 150 mM NaCl and 0.5% NP40, pH 7.6) for immuno-precipitation, as we have described below and previously [17].

### 2.6. Click chemistry

Click chemistry was performed as we have previously described [17]. Briefly, DiaAlk-labeled proteins were incubated with 100 μM Biotin-azide, 1 mM tris(2-carboxyethyl) phosphine hydrochloride (TCEP-HCL), 100 μM tris[(1-benzyl-1 H-1,2,3-triazol-4-yl) methyl] amine ligand (TBTA) and 1 mM CuSO_4_ for 1 h at room temperature with constant agitation. Reactions were quenched with 40 mM EDTA, proteins were precipitated by methanol-chloroform as described above, and lysates were probed using streptavidin-HRP antibody via immunoblotting, as we have described below and previously [17,19,28,33,34].

### 2.7. Immunoprecipitation of β_2_AR

Prepared lysates were tumbled with 10 μl (0.2 μg/μl) anti-human β_2_AR mouse monoclonal antibody (E3, Santa Cruz Biotechnology) for 2 h at 4 °C and 20 μl of resuspended protein A/G -agarose beads (Thermo Fischer Scientific) were added and tumbled overnight at 4 °C. Beads were washed by centrifugation three times with iced PBS, eluted with 1X Laemmli sample buffer with 2.5% β-mercaptoethanol for 20 min at room temperature and resolved by SDS-PAGE.

### 2.8. Immunoblotting

Immunoblotting was performed as we have described previously [17,19,28,33,34]. Briefly, after lysis in RIPA, protein concentrations were standardized using DC Protein Assay (Bio-Rad, Hercules, CA) and denatured in Laemmli sample buffer with 2.5% β-mercaptoethanol prior to resolution with SDS-PAGE. For analysis of ACV/VI and PDE4A expression, samples were boiled for 2 min, whereas for SOD, catalase, Gαs and Gαi-1/2, samples were boiled for 5 min, and β_2_AR samples were denatured at room temperature for 20 min. Equivalent concentrations of protein were resolved by SDS-PAGE and transferred to PVDF membrane. Blots were blocked in either 5% BSA or 3% non-fat milk solution (1X TBST) based on the appropriate primary antibody. Membranes were washed five-times and incubated with primary antibody for 1 h at room temperature or overnight at 4 °C. Blots were visualized using HRP-conjugated secondary antibody by ECL. Where blots were reprobed with another antibody, blots were stripped in 25 mM glycine for 45 min at 50 °C with constant agitation and re-probed following blocking. The following antibodies and respective concentrations were utilized for immunoblotting: β_2_AR H73 (1:1000, SC-9042), ACV-VI (1:1000, SC-514785), PDE4A (1:1000, SC-74428) and β-actin (1:1000, SC-47778) were obtained from Santa Cruz Biotechnology (Dallas, TX). Gαs (1:1000, 06–237), Gαi1/2 (1:1000, 06–236) were from Sigma Aldrich (St. Louis, MO). Catalase (1:1000, 12980), SOD1 (1:1000, 37385), and SOD2 (1:1000, 13141) were from Cell Signaling Technology (Danvers, MA), while SOD3 (1:1000, PIPA559870 was from ThermoFisher.

### 2.9. Quantification and statistical analysis

All the graphical data were created and analyzed using GraphPad Prism (La Jolla, CA) and represented as a mean ± standard deviation (SD). The means from each individual experiment performed in triplicate were pooled and the number of independent replicates is shown in figure legends. Where not visible, the error bars fall within the symbol size. Statistical analysis was performed with ninety-five percent confidence interval using one-way or two-way analysis of variance (ANOVA) and Tukey’s post-hoc analysis, as described in the figure legends. Statistical significance is represented as * *p* < 0.05, * * *p* < 0.01, * ** *p* < 0.001 and similar classification with the # symbol for second comparison, as noted in the figure legends. Values of *p* < 0.05 were defined a priori as statistically significant and reported *p* values are accompanied by Cohen’s *d* value, where appropriate, as a measure of the effect size to convey practical significance.

## 3. Results

### 3.1. Agonism of β2AR induces ROS generation in a receptor and NADPH oxidase-dependent manner in airway epithelial cells

**Fig. 1.**
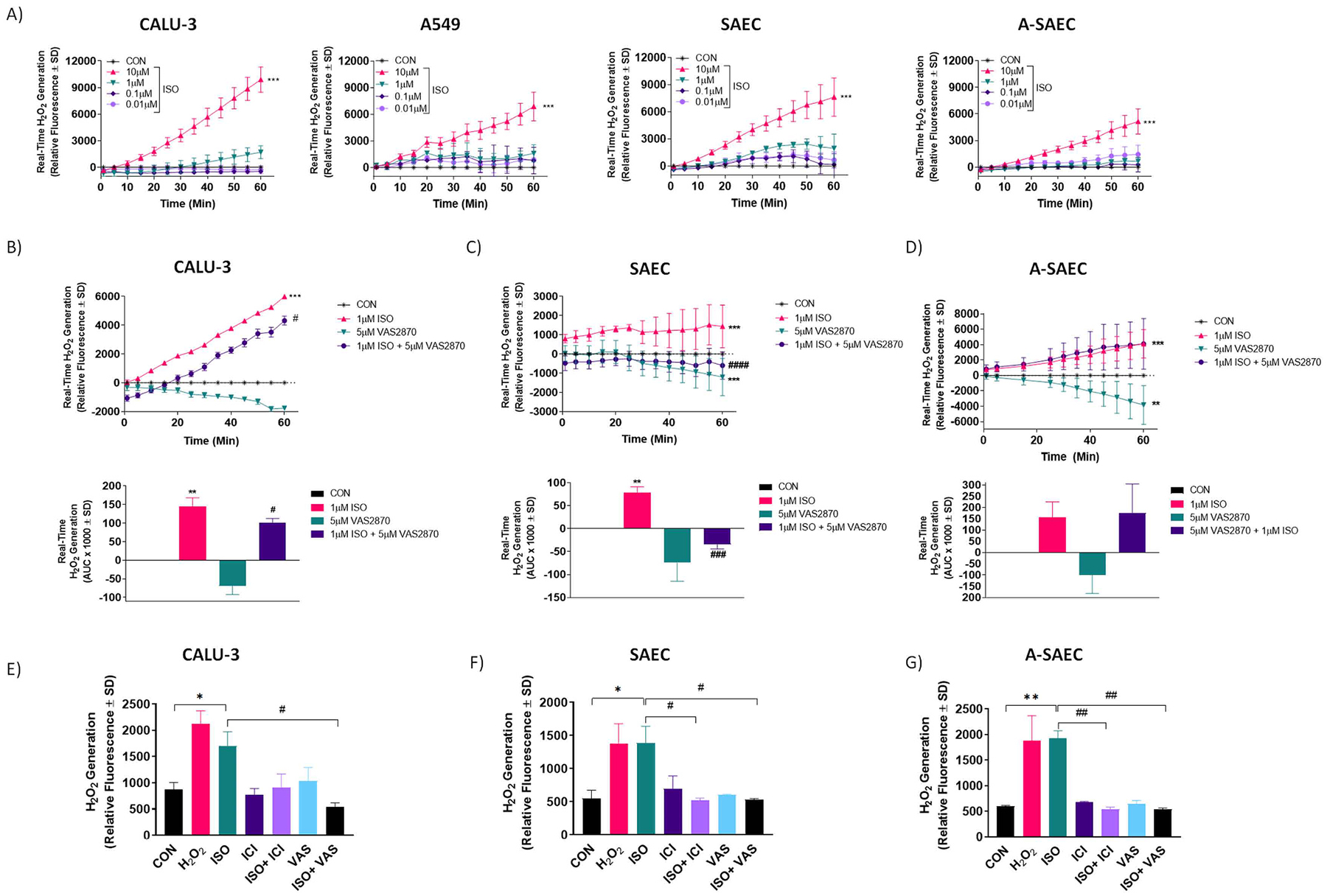
β2AR agonism stimulates H_2_O_2_ production in human airway epithelial cells in a NOX dependent manner. Agonism of β_2_AR with increasing ISO concentrations initiates concentration-dependent increases in real-time H_2_O_2_ production. **(A)** ISO (10 μM) significantly increases H_2_O_2_ production (*p* < 0.001 via One-way ANOVA with Tukey’s post-hoc) versus vehicle-control in CALU-3, A549, SAEC and A-SAEC, as detected by the extracellular H_2_O_2_-selective probe Amplex Red (*n* = 3). H_2_O_2_ (0.1 μM) was used as an internal positive control to ensure probe viability (data not shown). Statistical analysis was performed using one-way ANOVA with Tukey’s post-hoc test and * ** denotes *p* < 0.001 versus the vehicle-control condition. **(B-D)** ISO (1 μM) significantly increased (*p* < 0.001) H_2_O_2_ production in CALU3 **(B)**, SAEC **(C)** and A-SAEC **(D)** cells and pretreatment with the selective NOX inhibitor, VAS2870 (5 μM; 30 min) significantly attenuated real-time H_2_O_2_ generation in CALU-3 (*p* < 0.05 via ANOVA with Tukey’s post-hoc) and SAEC (*p* < 0.001 via ANOVA with Tukey’s post-hoc) (*n* = 3). The ISO-induced real-time H_2_O_2_ generation remained unaltered in the presence of VAS2870 in A-SAEC (*n* = 3). Compared to the ISO-alone condition, AUC analysis reveals a 30.7% and 148% reduction in ISO-induced real-time H_2_O_2_ generation in the presence of VAS2870 in CALU-3 (B, lower), and SAEC (C, lower), respectively (*n* = 3). Values represent average of three independent experiments (*n* = 3), performed in triplicates, vehicle control (unstimulated) values were subtracted from each data point for baseline correction. Statistical analysis was performed using one-way ANOVA with Tukey’s post-hoc test and * * denotes *p* < 0.01, and * ** denotes *p* < 0.001 versus the vehicle-control condition, while ^#^ denotes *p* < 0.05, ^###^ denotes *p* < 0.001, and ^####^ denotes *p* < 0.0001 versus the respective ISO-treated condition. **(E)** ISO (10 μM) significantly increases H_2_O_2_ production versus vehicle-control in CALU-3, SAEC and A-SAEC, as detected by the intracellular H_2_O_2_-selective probe AbGreen. Cells were pretreated with VAS2870 (VAS; 5 μM) or the β_2_AR inverse agonist ICI-118,551 (ICI; 10 μM) for 30 min, both of which significantly inhibit fluorescent intensity, demonstrating β_2_AR- and NOX-dependent H_2_O_2_ generation. H_2_O_2_ (0.1 μM) served as positive control in each experiment. Graphs represent quantified fluorescent intensity from 4X images from three independent experiments (*n* = 3) performed in triplicates. Statistical analysis was performed using one-way ANOVA with Tukey’s post-hoc test and * denotes *p* < 0.05, and * * denotes *p* < 0.01 versus the vehicle-control condition, while ^#^ denotes *p* < 0.05, and ^##^ denotes *p* < 0.01 versus the respective ISO-treated condition, as shown.

Previously, we have shown that agonism of β2AR with the β-receptor agonist isoproterenol (ISO) generates ROS in clonal cell lines as well as in human airway cells [17–20]. Similar results have been shown by others in a variety of cells and tissues [21–23,25,35,36]. Here, for the first time, we examine real-time β2AR-mediated H_2_O_2_ generation in human airway epithelial cell lines A549 and CALU-3, as well as in primary human small airway epithelial cells from healthy and asthma-diseased patients. Since airway epithelial cells have been shown to consist almost entirely of the β2-subtype [37,38], we used the full-efficacy β-receptor agonist ISO in a fluorescent-based assay that utilizes the highly sensitive, extracellular Amplex Red probe that detects H_2_O_2_ in real-time. Similar to our previous results with non-specific ROS probes [18,20], our results here show that ISO concentration-dependently induces real-time H_2_O_2_ generation in CALU-3 and A549 human airway epithelial cell lines, validating the H_2_O_2_-specific probe (Fig. 1A). These effects were most detectable at concentrations of 1 μM or higher, as reported by us and others in a variety of cell and tissue types [18–23,25,28,35,36]. The ISO-induced H_2_O_2_ generation was also seen to the same degree in non-diseased SAEC from a healthy human donor, and responded similarly as the airway cell lines to treatment with ISO, while asthma-diseased SAEC from an asthmatic patient (A-SAEC) exhibited a lower level of H_2_O_2_ in response to 10 μM ISO than CALU-3, A549 or SAEC (Fig. 1A) H_2_O_2_ at a concentration of 0.1 μM was used as an internal positive control, and produced an equivalent signal as the highest concentration of ISO used here (data not shown).

We have previously demonstrated that β2AR-linked ROS generation in airway epithelial cell lines is coupled to activation of NADPH oxidase (NOX) isoforms [18–20]. To assess this effect in SAEC and A-SAEC, we utilized the selective NOX inhibitor VAS2870 along with the 1 μM concentration of ISO in the real-time measure of H_2_O_2_ generation with Amplex Red. In CALU-3 airway epithelial cells, VAS2870 (5 μM) decreased basal H_2_O_2_ generation over 60 min, consistent with inhibition of NOX (Fig. 1B, upper). In the presence of VAS2780, ISO-induced (1 μM) H_2_O_2_ generation was significantly (*p* < 0.05) right-shifted, and decreased at each time point compared to ISO alone (Fig. 1B, upper). Since this is a real-time, rather than an endpoint measurement, we quantified the area under the curve (AUC) of these effects over 60 min and our results showed a 30.7% reduction of ISO-induced H_2_O_2_ generation in the presence of VAS2870 (Fig. 1B, lower) in CALU-3 cells. In healthy SAEC, VAS2870 alone (5 μM) also decreased basal H_2_O_2_ generation over 60 min, however unlike CALU-3 cells, in the presence of VAS2780, ISO-induced H_2_O_2_ generation in SAEC was fully inhibited (p < 0.001) (Fig. 1C, upper). Analysis of AUC in SAEC revealed a 148% decrease in ISO-induced H_2_O_2_ generation in the presence of VAS2870 (Fig. 1C, lower). On the contrary, and to our surprise, the effects of ISO on real-time H_2_O_2_ generation in asthma-diseased SAEC were unaltered by the presence of VAS2870, even though the NOX inhibitor reduced basal H_2_O_2_ generation when used alone (Fig. 1D). To our knowledge, these are the first results that link β2AR agonism to H_2_O_2_ generation in primary healthy and asthma-diseased SAEC.

Since Amplex Red is cell-impermeable and detects only extracellular H_2_O_2_, we also wished to utilize a cell-permeable H_2_O_2_ probe that is sensitive to intracellular H_2_O_2_ and hence, we used a distinct H_2_O_2_-selective fluorescent probe for detection of intracellular H_2_O_2_, similar to that which we and others have reported on previously [18,20,27,39]. Since we have utilized this approach for detection of intracellular ROS in CALU-3 cells previously [18], this cell model was used as a positive control here and again demonstrated robust fluorescence, indicative of intracellular H_2_O_2_ generation, upon agonism with ISO (10 μM) and the scale of oxidant generation here was on par with 0.1 μM H_2_O_2_ (Fig. 1E–G). Quantification of fluorescence demonstrates approximately 1.5–2–fold increase in H_2_O_2_ generation upon agonism with ISO, consistent with ours and other’s previous reports on the scale (1.25–3–fold over basal) of β2AR-induced ROS generation [18,19,27, 40]. In CALU-3 cells, the ISO-induced effect was abolished by the selective β2-receptor antagonist ICI-118,551 (10 μM) as well as the selective NADPH oxidase inhibitor VAS2870 (5 μM), confirming our previous and above results [18] that show that ISO-induced H_2_O_2_ generation in airway epithelial cells is β2AR and NOX dependent (Fig. 1E). Importantly, our results reveal that ISO induces robust H_2_O_2_ generation of a similar magnitude seen in CALU-3 cells in both SAEC and A-SAEC, and these effects were significantly inhibited by both ICI-118,551 and VAS2870, demonstrating that β2AR agonism can facilitate H_2_O_2_ generation in primary SAEC in a manner dependent on NADPH oxidase isoforms, presumably from dismutation of superoxide via SOD or directly via NOX4 (Fig. 1F–G). Interestingly, this result from A-SAEC is contrary to those in Fig. 1D that show a lack of effect of VAS2870 in A-SAEC when the extracellular H_2_O_2_ probe is used, and suggest differences in A-SAEC that mitigate extracellular presence of H_2_O_2_.

### 3.2. H_2_O_2_ and receptor agonism facilitates cysteine S-sulfination of β2AR

**Fig. 2.**
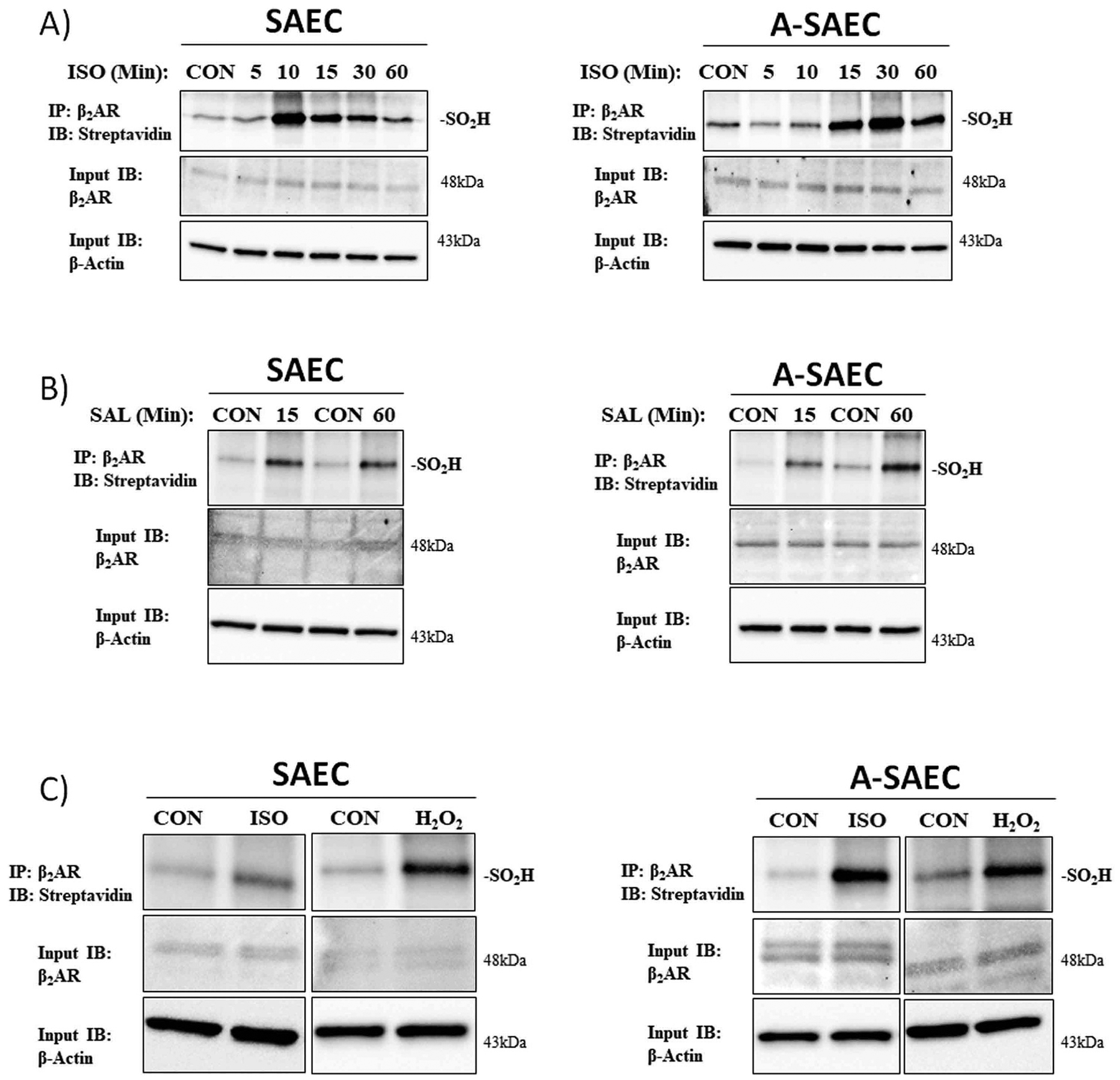
Exogenous H2O_2_ and β-receptor agonism induces β_2_AR Cys-S-sulfination in human SAEC. **(A)** The ability of acute agonism of β2AR to induce cysteine-S-sulfination was assessed via the clickable S-sulfinic acid-selective probe DiaAlk. SAEC or A-SAEC (4 ×10^5^ cells/well) were seeded in 6-well plates and agonized with **(A)** ISO (10 μM) for 5–60 min (*n* = 3) or **(B)** salbutamol (albuterol; SAL) (10 μM) for 15 or 60 min (*n* = 3). β2AR cysteine-S-sulfination was assessed by β2AR immunoprecipitation and selective labeling with 1 mM DiaAlk, followed by conjugated with biotin utilizing click chemistry, and detection via streptavidin-HRP, as discussed in the materials and methods. Both ISO **(A)** and SAL **(B)** significantly increased β_2_AR hyperoxidation to cysteine-S-sulfinic acids (upper panels). The amount of β2AR (middle panels) within each reaction is shown as an input control and β-actin (lower panels) is utilized to denote equal protein input and loading. **(C)** The effects of chronic ISO agonism or H_2_O_2_ were also assessed and cells were treated every 12 h with for seven days with either ISO (10 μM) or H_2_O_2_ (10 μM). The media was replenished every 2–3 days to prevent nutrient depletion-mediated starvation. Both SAEC and A-SAEC exhibit significantly elevated DiaAlk-biotin labeled β2AR (upper panels), indicative of cysteine-S-sulfination, following the seven-day treatment paradigm with both ISO and H_2_O_2_. The amount of β2AR (middle panels) within each reaction is shown as an input control and β-actin (lower panels) is utilized to denote equal protein input and loading.

We have previously reported that β2AR cysteine residues can be oxidized by ROS, including H_2_O_2_, to form cysteine-S-sulfenic acids (Cys-SOH), and this effect is also mediated by ROS generated upon agonism of β2AR [18,28]. Importantly, transient β2AR S-sulfenation is required for proper receptor function while inhibition of the recycling of -SOH groups back to the reduced thiol (-SH) inhibits proper receptor signaling [18]. Moreover, our previous results showed that ‘trapping’ of S-sulfenic acids by the selective sulfenic acid alkylator dimedone, which mimics higher-order S-sulfinic/S-sulfonic acids by irreversibly preventing normal redox recycling, abolishes β2AR signaling [18]. Based on these results, we hypothesized that in the presence of millimolar concentrations of H_2_O_2_, β2AR may be overoxidized from cysteine S-sulfenic acids to higher order S-sulfinic and/or S-sulfonic acids, which are irreversible and would compromise receptor function. To determine if receptor agonism and exogenous H_2_O_2_ induce formation of β2AR-Cys-S-sulfinic acids in SAEC and A-SAEC, we developed an immunoblot-based assay that makes use of the clickable selective S-sulfinic acid probe DiAlk, which does not recognize S-sulfenic or S-sulfonic acids [41–44]. Preliminary experiments show that treatment of cells with 1 mM H_2_O_2_ induced cysteine-S-sulfination in a time-dependent manner (data not shown). Acute treatment of both SAEC and A-SAEC with ISO over a time period from five to sixty min resulted in significant S-sulfination of β2AR compared to vehicle-treated control (Fig. 2A). We also assessed β2AR S-sulfination upon agonism with salbutamol (also referred to as albuterol), a β2-selective partial agonist that is functionally biased toward β2AR-Gαs over β-arrestin signaling [45–47], and is heavily used in the clinical treatment of asthma. Agonism of β2AR with salbutamol for 15 and 60 min also resulted in significantly elevated β2AR S-sulfination compared to vehicle-treated controls in both SAEC and A-SAEC cells (Fig. 2B). Next, we determined the effects of chronic ISO and H_2_O_2_ treatment on S-sulfination of β2AR. SAEC and A-SAEC were treated with ISO (10 μM) or H_2_O_2_ (10 μM) every twelve hours for seven days and subjected to the DiaAlk labeling procedure described above to detect S-sulfinated β2AR. Our results show that chronic treatment with ISO and H_2_O_2_ in this paradigm facilitated significantly higher S-sulfinated β2AR than vehicle-treated control conditions (Fig. 2C). Taken together, our results demonstrate for the first time that β2-agonism as well as millimolar H_2_O_2_ can facilitate irreversible S-sulfinic acid oxidation of β2AR.

### 3.3. H_2_O_2_ alters β2AR-induced cyclic AMP signaling

**Fig. 3.**
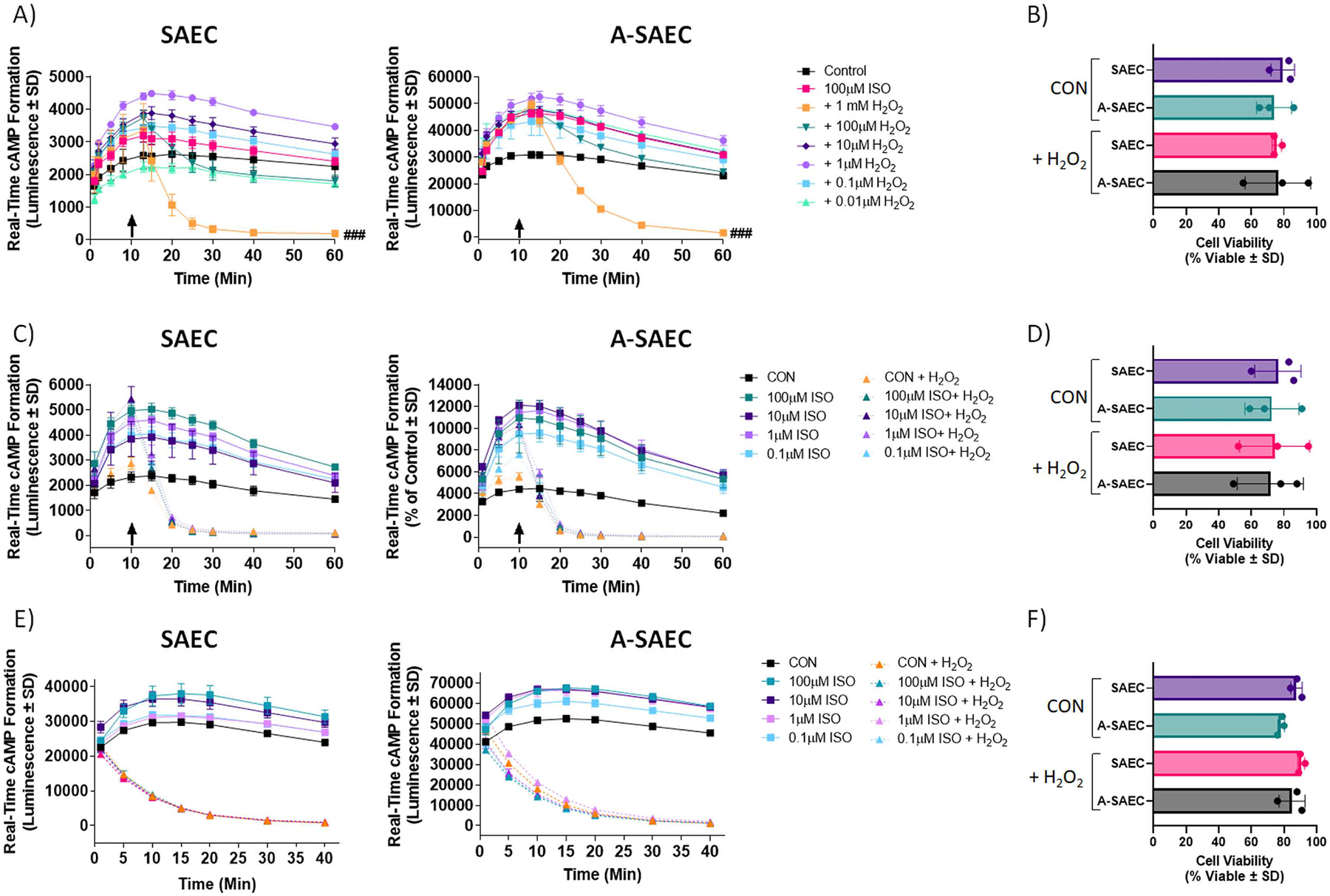
Low concentrations of exogenous H2O_2_ enhance, while 1 mM H_2_O_2_ inhibits ISO-induced cAMP production. **(A)** Real-time cAMP was assessed in the presence of various H_2_O_2_ concentrations introduced 10 min (arrows) following agonism with ISO. While H_2_O_2_ concentrations spanning 0.1–1 μM enhanced cAMP production, higher concentrations decreased it, and the 1 mM H_2_O_2_ concentration significantly inhibited ISO-induced cAMP production in both **(A)** SAEC (*p* < 0.001) and A-SAEC (*p* < 0.001). Statistical analysis was performed using one-way ANOVA with Tukey’s post-hoc test and ^###^ denotes *p* < 0.001 versus the respective ISO-treated condition (*n* = 3). **(B)** To ensure that the effect of 1 mM H_2_O_2_ was not due to induction of cell death, cell viability was assessed 4 h following agonism with ISO via trypan blue exclusion and there was no significant effect of the 1 mM H_2_O_2_ treatment on cell viability in either SAEC or A-SAEC (pooled data from *n* = 3). **(C)** The effects of 1 mM H_2_O_2_ were also assessed on variable ISO concentrations (0.1 μM - 100 μM) and in both SAEC and A-SAEC, 1 mM H_2_O_2_ added 10 min (arrows) following agonism with ISO abolished the cAMP-generating effects of all concentrations of ISO (*p* < 0.0001 for both cell types, one-way ANOVA, *n* = 3), and this effect remained for the 4 h observation duration (not shown). **(D)** To ensure that the effect of 1 mM H_2_O_2_ was not due to induction of cell death, cell viability was assessed 4 h following agonism with ISO via trypan blue exclusion and there was no significant effect of the 1 mM H_2_O_2_ treatment on cell viability in either SAEC or A-SAEC (pooled data from *n* = 3). **(E)** Given that these results reflect effects of H_2_O_2_ treatment following agonism with ISO, we also assessed the outcomes of ISO-induced cAMP formation in cells pre-incubated with H_2_O_2_ (1 mM) for 1 min prior to agonism with ISO, as we previously showed this was enough to induce cysteine-S-sulfenation of β2AR [18,28]. In this case, H_2_O_2_ also significantly inhibited cAMP formation in both SAEC (*p* < 0.0001) and A-SAEC (*p* < 0.0001), with no significant effect on cell viability **(F)** (*n* = 3).

Our previous work has shown that oxidation of β2AR to S-sulfenic (SOH) acids enhances receptor activity, while irreversible modification of β2AR cysteine-S-sulfenic (SOH) acids, which prevents their normal recycling back to the free thiol (SH), inhibits proper receptor function [18, 28]. Our results above show that β2AR can also be oxidized to yield higher order cysteine S-sulfinic (SO_2_H) acid modifications, which are known to be generally irreversible. Given the currently accepted paradigm that micromolar H_2_O_2_ concentrations regulate homeostatic signaling, while higher concentrations (e.g., millimolar) facilitate unfavorable oxidative stress responses, including irreversible post-translational protein oxomodification to cysteine-S-sulfinic/sulfonic acids [48–53], we wished to assess the effects of heightened H_2_O_2_ on β2AR signaling. To begin to do so, we used a biosensor-based real-time cAMP formation assay to measure ISO-induced cAMP formation over a 4 h time-frame. Cells were treated with a saturating concentration of ISO (100 μM) and after 10 min were treated with varying concentrations of H_2_O_2_. As shown in Fig. 3A, addition of 0.1–1 μM concentrations of H_2_O_2_ slightly enhanced ISO-induced cAMP formation in both healthy and asthma-diseased SAEC, consistent with our previous results demonstrating that S-sulfenation of β2AR enhances receptor activity [18]. On the contrary, addition of 1 mM H_2_O_2_ lead to a significant decrease in ISO-induced cAMP formation that lasted the duration of the 4 h observation period shown in both SAEC and A-SAEC (Fig. 3A) (for clarity only the first 60 min is shown). To ensure that the noted effect of 1 mM H_2_O_2_ was not a byproduct of cell death, we assessed cell viability of SAEC and A-SAEC in the absence and presence of H_2_O_2_ over the 4 h time-frame and results show no significant differences in viability, with all conditions exhibiting approximately 80% viability after 4 h (Fig. 3B). Since millimolar concentrations of H_2_O_2_ seemed to effectively inhibit β2AR agonism, we also assessed the effects of the oxidant in the presence of variable concentrations of agonist (0.1–100 μM). Results in Fig. 3C demonstrate that addition of 1 mM H_2_O_2_ 10 min following agonism with ISO decreases both basal and ISO-stimulated cAMP formation at all concentrations of the agonist tested. In both cases, addition of the oxidant steadily decreased cAMP formation for approximately 10 additional min and cAMP levels were sustained at this level (Fig. 3A, C) for up to 4 h (data not shown). Importantly, as our results in Fig. 5 will show, β2AR is responsible for a significant level of constitutive, agonist-independent cAMP formation in these cells, hence the decrease in the basal cAMP seen here upon H_2_O_2_ treatment likely includes effects on β2AR-mediated basal cAMP formation. Again, to ensure this effect of H_2_O_2_ was not due to cell death, we performed cell viability assays and results show no significant differences in cell viability in the absence or presence of H_2_O_2_ at the end of the observation period (Fig. 3D).

Previously, we have shown that β2AR can be oxidized to S-sulfenic acids as quickly as one min following addition of H_2_O_2_ [18,28]. Next, we assessed whether H_2_O_2_ (1 mM) treatment prior to agonism with ISO would similarly affect cAMP formation. Both SAEC and A-SAEC were treated with 1 mM H_2_O_2_ one min prior to treatment with variable concentrations (0.1–100 μM) of ISO in the absence or presence of the oxidant. As shown in Fig. 3E, ISO concentration-dependently increased cAMP in both SAEC and A-SAEC, however, pretreatment with 1 mM H_2_O_2_ prevented this increase at all ISO concentrations and in both cell types (up to 40 min shown). Cell viability assays again showed no significant differences in cell viability in the absence or presence of H_2_O_2_ at the end of the 4 h observation period (Fig. 3F). We also wished to ensure that the results seen here were not due to direct effects of H_2_O_2_ on the real-time luminescent cAMP biosensor used in our assays. Hence, we restimulated treated cells at 230 min following ISO addition with the adenylyl cyclase (AC) activator forskolin (FSK; 100 μM) and monitored cAMP for an additional 10 min, which yielded an increase of 2–3–fold in cAMP formation, demonstrating that the cAMP biosensor was still active (data not shown). Our data demonstrate that while lower H_2_O_2_ levels enhance β2AR-mediated cAMP formation, likely via S-sulfenation, high concentrations of H_2_O_2_ (e.g., 1 mM) can dramatically inhibit, or prevent, ISO-stimulated cAMP formation, and that this effect is not due to cell death, rather, we presume, by irreversible S-sulfination of β2AR.

**Fig. 4.**
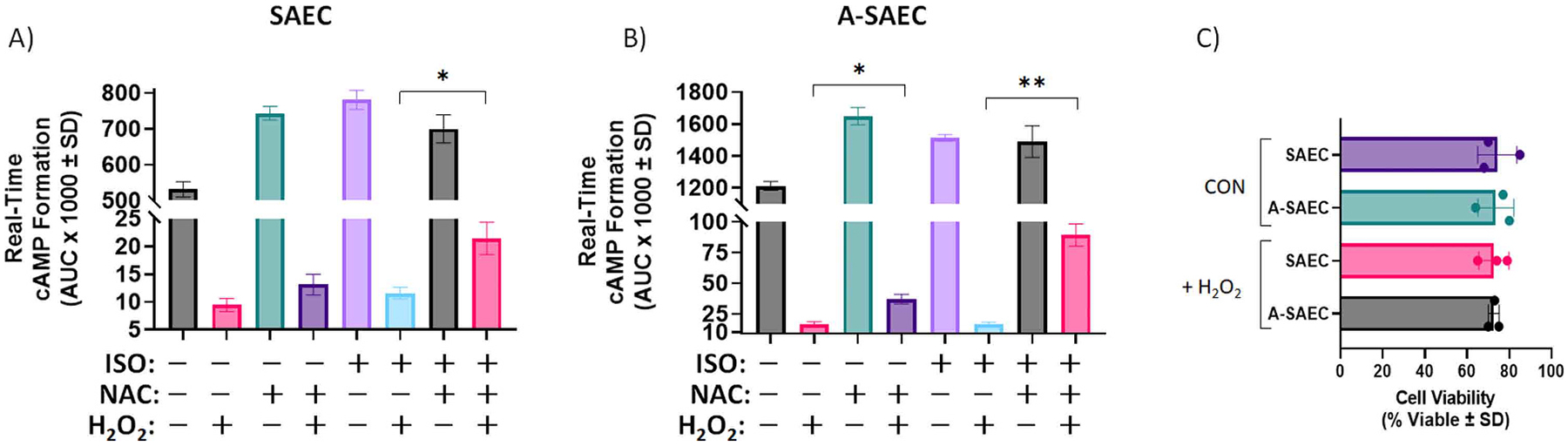
The antioxidant and cysteine-donor *N*-acetyl-*L*-cysteine restores the H_2_O_2_-ablated ISO-induced cAMP response. **(A-B)** *N*-acetyl-_*L*_-cysteine (NAC; 1 mM) treatment reverses the inhibitory effect of 1 mM H_2_O_2_ on ISO-induced cAMP formation in both SAEC and A-SAEC. AUC analysis from real-time cAMP measurements from 40 to 200 min following agonism reveals significantly increased ISO-induced cAMP response in **(A)** healthy SAEC (*p* < 0.05 via ANOVA with Tukey’s post-hoc) and **(B)** asthmatic SAECs (*p* < 0.01 via ANOVA with Tukey’s post-hoc), treated with H_2_O_2_, compared to the H_2_O_2_ alone-treated condition. In addition, NAC significantly enhanced basal cAMP levels after H_2_O_2_ treatment in A-SAEC (*p* < 0.05 via ANOVA with Tukey’s post-hoc), compared to healthy SAEC (*n* = 3). **(C)** To ensure that the effect of 1 mM H_2_O_2_ was not due to induction of cell death, cell viability was assessed 4 h following agonism with ISO via trypan blue exclusion and there was no significant effect of the 1 mM H_2_O_2_ treatment on cell viability in either SAEC or A-SAEC (pooled data from *n* = 3). Statistical analysis was performed using one-way ANOVA with Tukey’s post-hoc test and * denotes *p* < 0.05, while * * denotes *p* < 0.01, as shown.

Next, we wished to determine if the ROS sequestering antioxidant *N*-acetyl-_*L*_-cysteine (NAC), which acts as a cysteine martyr to scavenge H_2_O_2_, and hence prevent its ability to oxidize other cysteines, would alter the H_2_O_2_ induced effect on ISO-mediated cAMP formation. Here, we quantified the area under the curves from the 40–200 min timeframe for each real-time cAMP condition, a point at which the cAMP reduction was at the fully sustained nadir by H_2_O_2_ (Fig. 3A, C, E), in the absence and presence of NAC (1 mM). In SAEC, treatment with NAC slightly but non-significantly increased basal cAMP concentrations following reduction by H_2_O_2_ (Fig. 4A), while the effect of NAC on ISO-mediated cAMP formation was significantly pronounced compared to the ISO-alone condition in the presence of H_2_O_2_ (Fig. 4A). This result suggests that NAC specifically protects β2AR from the effects of the oxidant. These effects were also demonstrated in A-SAEC, which demonstrated higher basal cAMP formation when NAC was in the presence of H_2_O_2_ and also exhibited higher ISO-induced cAMP formation in the presence of NAC compared to that seen with ISO alone (Fig. 4B). Cell viability was again similar for all conditions (Fig. 4C). Together, these results show that H_2_O_2_ can significantly influence β2AR signaling, and that cysteine-donating antioxidants can reverse the negative effect of high H_2_O_2_ concentrations, at least to a degree.

### 3.4. β2AR-mediated cAMP formation is altered in normal versus asthmatic SAEC

**Fig. 5.**
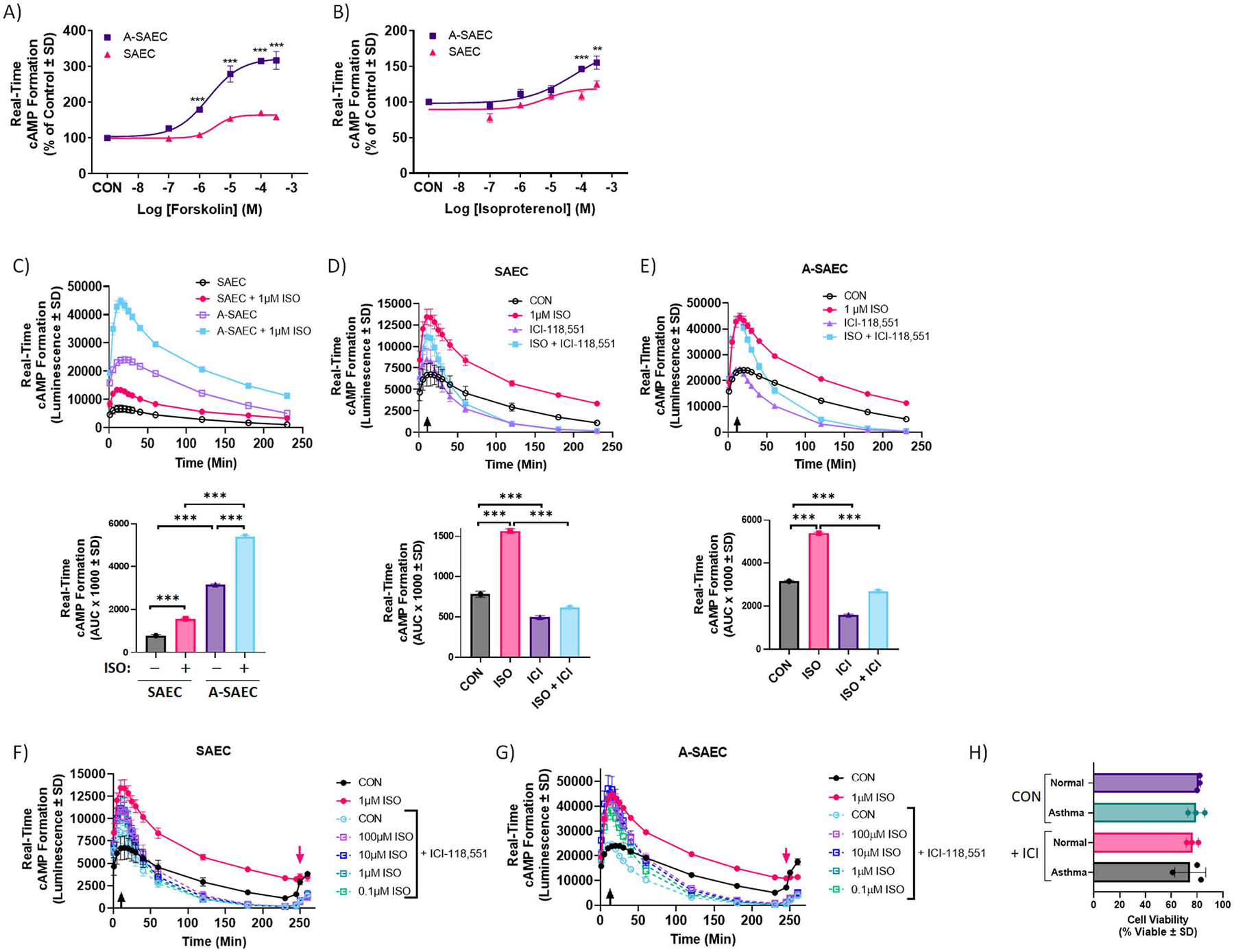
Altered real-time β2AR-mediated cAMP formation in SAECs. **(A-B)** Asthma-derived SAEC exhibit significantly higher cAMP formation (*p* < 0.001) compared to healthy SAEC upon stimulation with **(A)** forskolin (FSK) or **(B)** ISO. Each point in the graph represents mean of 3 independent experiments performed in triplicates (*n* = 3). Statistical analysis was performed using one-way ANOVA with Tukey’s post-hoc test and significance denoted as * * *p* < 0.01 or * ** *p* < 0.001 versus healthy SAEC. Given the variable concentrations of cAMP in each cell type, data are normalized to a percentage of the unstimulated SAEC response ± SD. **(C, upper)** Over a 4 h time period, both basal and ISO-induced (1 μM) real-time cAMP formation were significantly elevated in A-SAEC compared to healthy SAEC (*p* < 0.001, two-way ANOVA). **(C, lower)** AUC analysis over the entire time-frame also revealed significantly higher real-time cAMP formation in A-SAEC vs SAEC (*p* < 0.001, two-way ANOVA). **(D-E)** Agonism of β_2_AR with ISO (1 μM) induces significant increases in real-time cAMP formation in **(D)** SAEC and **(E)** A-SAEC, and this effect was abolished (*p* < 0.0001) in the presence of β_2_AR inverse agonist, ICI-118,551 (100 μM) introduced 10 min (arrows) following agonism with ISO, indicating that the effects seen are β_2_AR specific. Notably, basal cAMP formation in the absence of agonist was also significantly reduced by ICI-118,551 treatment, demonstrating a significant contribution of β2AR to constitutive cAMP signaling in both SAEC and A-SAEC. **(D-E, lower)** AUC analysis over the entire time-frame also revealed the effects of ICI-118,551 alone and with ISO in A-SAEC vs SAEC. Statistical analysis was performed using one-way ANOVA with Tukey’s post-hoc test and significance denoted as * ** *p* < 0.001, as shown. **(F-G)** ICI-118,551 treatment 10 min (black arrows) following agonism with ISO blocks both basal and ISO effects at various concentrations of ISO (0.1–100 μM) in both **(F)** SAEC and **(G)** A-SAEC. To ensure that cells were viable after 4 h, they were re-challenged with 10 μM FSK/100 μM IBMX (red arrows), and responded with increases in real-time cAMP generation. **(H)** To confirm that the effects of ICI-118,551 were not due to induction of cell death, cell viability was assessed 4 h following agonism with ISO via trypan blue exclusion and there was no significant effect of ISO-118,551 treatment on cell viability in either SAEC or A-SAEC (pooled data from *n* = 3).

Our results in Fig. 3 bring to light significant elevations in both basal and agonist-induced cAMP formation in asthma-diseased SAEC compared to healthy cells (Fig. 3A, C, E). To investigate this difference further, we assessed the effects of ISO and FSK in real-time cAMP assays in SAEC and A-SAEC. Notably, as typically performed in cAMP assays, the PDE inhibitor IBMX is utilized here to inhibit turnover of cAMP. Consistent with ISO results seen in Fig. 3, FSK produced significantly higher concentration-dependent cAMP formation in asthma-diseased cells compared to normal SAEC at concentrations of 1, 10, 100, and 300 μM (p < 0.001 for each versus normal, d = 11.8, 4.4, 19.3, and 5.5 versus normal, respectively), suggesting differences are not β2AR mediated (Fig. 5A). Compared to FSK, agonism of healthy SAEC with ISO yielded a predictably smaller concentration-dependent increase in cAMP formation that peaked at approximately 125% of untreated control, but was again significantly higher in asthma-diseased compared to normal SAEC at ISO concentrations of 100 μM and 300 μM (p < 0.001 and 0.01 versus normal, d = 2.4 and 5.0, respectively) (Fig. 5B).

We then probed ISO-stimulated real-time cAMP generation in SAEC and A-SAEC over a 4 h time frame using 1 μM concentrations of the agonist, and our results demonstrate clear and profound differences in both basal (i.e., vehicle) and ISO-induced cAMP formation between SAEC and A-SAEC (Fig. 5C, upper). Two-way ANOVA with Tukey’s multiple comparisons test revealed significant differences between SAEC and A-SAEC (*p* < 0.0001) as well as between the vehicle and ISO-treated groups within each cell type (*p* < 0.0001). Given the real-time nature of this series of experiments, we assessed area under the curve (AUC) analysis, which showed significant increases in total real-time cAMP in ISO treated conditions compared to vehicle-treated conditions for both SAEC and A-SAEC (*p* < 0.0001, one-way ANOVA) (Fig. 5C, lower). Moreover, A-SAEC exhibited significantly higher basal (*p* < 0.0001) and ISO-induced (*p* < 0.0001) cAMP formation compared to SAEC (Fig. 5C upper and lower).

To ensure that the effects of ISO on real-time cAMP formation were mediated by β2AR, we utilized the selective β2AR inverse-agonist ICI-118,551 in the absence or presence of ISO (1 μM). Given that this is a real-time assay, ISO was added first and a saturating concentration of ICI-118,551 (100 μM) was added ten min after ISO addition. Importantly, we utilized a high concentration of the antagonist in these assays due to the real-time nature and high-sensitivity of this assay, as well as initial treatment with ISO and presence of IBMX, that allows for the sustained detection of luminescence from any cAMP generated upon ISO-induced agonism. In SAEC, ICI-118,551 significantly decreased basal cAMP formation and both left-shifted and significantly decreased the real-time ISO-mediated cAMP formation, demonstrating the β2AR dependent effect of ISO in these cells (Fig. 5D, upper). Two-way ANOVA test revealed significant differences between time, ISO versus control, ICI-118,551 versus control, and the ISO + ICI condition (*p* < 0.0001) (Fig. 5D, upper). AUC analysis showed significant increases in total real-time cAMP in the ISO treated condition compared to vehicle-treated (*p* < 0.0001, one-way ANOVA), and this effect was significantly blocked by ICI-118,551 (*p* < 0.0001, one-way ANOVA), which also decreased basal cAMP formation versus vehicle-treated (*p* < 0.0001, one-way ANOVA) (Fig. 5D, lower).

The effect of ISO was similar in A-SAEC, although the magnitude of basal and ISO-stimulated real-time cAMP formation was significantly higher in diseased cells compared to normal SAEC, as described above (Fig. 5E). Two-way ANOVA analysis again showed significant differences between time, ISO versus control, ICI-118,551 versus control, and the ISO + ICI condition (*p* < 0.0001) (Fig. 5E, upper). Similarly, AUC analysis showed significant increases in total real-time cAMP in the ISO treated condition compared to vehicle-treated (*p* < 0.0001, one-way ANOVA), and this effect was significantly blocked by ICI-118,551 (*p* < 0.0001, one-way ANOVA), which also decreased basal cAMP formation versus vehicle-treated (*p* < 0.0001, one-way ANOVA) (Fig. 5E, lower). Of note, basal cAMP formation in both SAEC and A-SAEC (Fig. 5D–E) was significantly decreased in the presence of ICI-118,551 alone, demonstrating that constitutive β2AR activity makes a considerable contribution toward the basal cAMP in these cells, which importantly, was inhibited by 1 mM H_2_O_2_, as shown in Fig. 3C, E. These data suggest that the effects of 1 mM H_2_O_2_ on basal cAMP seen in Fig. 3 can at least in part be due to effects on β2AR contribution to this basal signal.

Agonism with a range of ISO (0.1–100 μM) produced similar concentration-dependent increases in cAMP formation in both SAEC and A-SAEC (data not shown), and ICI-118,551 blocked both basal and ISO effects at each of these concentrations in both normal and asthma-diseased cells (Fig. 5F–G), however, the magnitude of the ISO effect was again significantly enhanced in A-SAEC (Fig. 5F–G). To ensure that the time-dependent decrease in cAMP formation seen in these cells was not a byproduct of toxicity or cell death we detected cell viability at 4 h following the addition of ISO and results show that there were no differences in cell viability between SAEC and A-SAEC alone or upon treatment (Fig. 5H). Finally, to ensure that inhibitory effects of ICI-118,551 were not due to degradation of the cAMP biosensor over time, we stimulated the cells at the 4 h mark with IBMX and FSK (100 μM), which resulted in another increase in real-time cAMP formation in control SAEC and A-SAEC, with both increasing approximately 250% (red arrow, Fig. 5F–G). Notably, a small increase in cAMP appeared here even in ICI-118,551 treated cells, but not in cells treated with ISO alone (Fig. 5F–G), suggesting a saturation of the cAMP effect, perhaps due to agonist-induced desensitization, in these cells upon treatment with ISO. Together, these results show that asthma-diseased SAEC exhibit significantly higher basal and ISO-induced cAMP formation compared to the healthy SAEC.

### 3.5. Asthma-diseased SAEC exhibit lower adenylyl cyclase V/VI and enhanced PDE4 expression, but similar β2AR, Gαs, Gαi, SOD1–3 and catalase expression

**Fig. 6.**
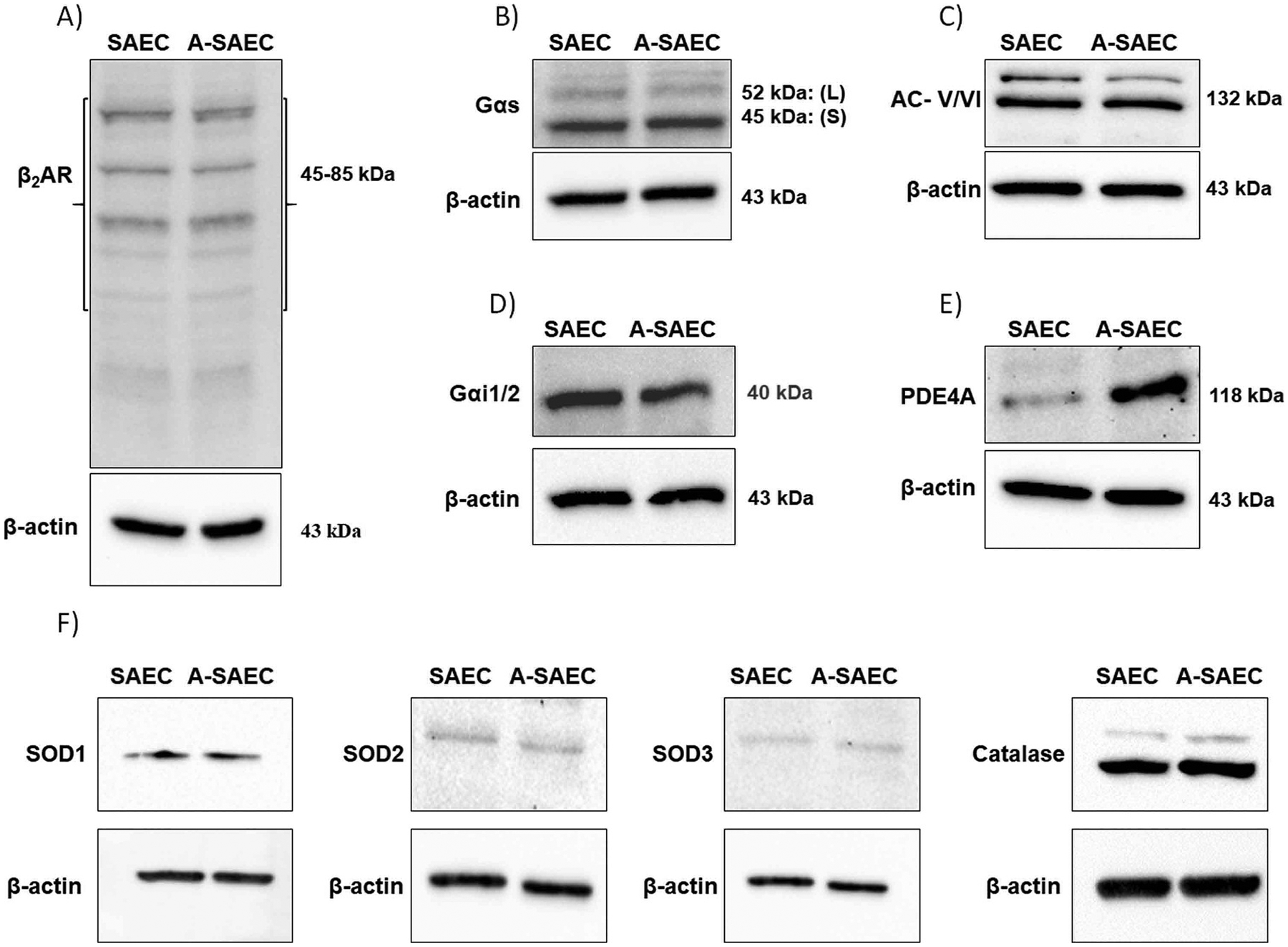
A-SAEC exhibit lower adenylyl cyclase V/VI and enhanced PDE4 expression, but similar β2AR, Gαs, and Gαi, SOD1–3, and catalase expression. Immunoblot analysis of SAEC and A-SAEC protein lysates for detection of proteins that could contribute to alterations in cAMP formation. Expression of **(A)** β_2_AR (56 – 85 kDa represents native and variably glycosylated β2AR species) and **(B)** Gαs (45 and 52 kDa bands represent short and long isoforms, respectively) were unaltered in SAEC compared to A-SAEC (*n* = 3, each). **(C)** Expression of ACV/VI was slightly decreased in A-SAEC compared to SAEC (*n* = 4), while expression of Gαi_1/2_ isoform (*n* = 3) was not significantly different **(D)**. **(E)** Expression of PDE4 was significantly heightened in A-SAEC compared to SAEC (*n* = 4). β-actin served as a loading control for each representative immunoblot. **(F)** Expression of SOD1, SOD2, SOD3, and was not significantly altered, while catalase expression was visually slightly, though not significantly, higher in A-SAEC. SOD2–3 were probed from the same blots, hence, actins are the same.

Next, we wished to investigate potential molecular mechanisms whereby asthma-diseased SAEC may exhibit the altered cAMP signaling that we observe in diseased versus healthy SAEC and to also probe the expression of H_2_O_2_-generating SOD isotypes and H_2_O_2_-degrading catalase. Since β2AR engages Gαs proteins to activate AC isoforms to generate cAMP, and given the role of ACV/VI in β2AR signaling in the lung [54,55], we first examined the expression of β2AR, Gαs, and ACV/VI in SAEC and A-SAEC. As revealed by immunoblotting, there were no notable differences in expression in β2AR or the long or short isoforms of Gαs, between SAEC or A-SAEC (Fig. 6A–B). Single point radioligand binding assays using a saturating concentration (12 nM) of [^3^H]-dihydroalprenolol confirmed that A-SAEC do not express greater numbers of β2AR (data not shown). However, we did note a subtle decrease in expression of ACV/VI in A-SAEC compared to healthy cells (Fig. 6C). Interestingly, this effect would be expected to lead to decreased, rather than increased, cAMP in A-SAEC. Since agonism of β2AR is known to be able to switch to coupling from Gαs to Gαi in a manner dependent on PKA-mediated receptor phosphorylation [56], we also examined whether the noted cAMP elevations observed in A-SAEC were due to impaired Gαi expression, however, our results indicate that expression of Gαi1/2 was not significantly different than that in SAEC (Fig. 6D). Finally, since phosphodiesterase isoforms, particularly PDE4A, play a significant role [57–61] in the regulation of cAMP levels in the airway epithelium, we examined differences in PDE4 expression in SAEC and A-SAEC. To our surprise, our results demonstrate a significant overexpression of PDE4 in asthma-diseased cells compared to normal SAEC (Fig. 6E). Again, this effect would be expected to lead to significantly lower, rather than elevated cAMP in A-SAEC, and are contrary to our cAMP data. These results indicate the asthma-diseased SAEC express subtly less ACV/VI and significantly higher PDE4, contrary to our results showing significantly elevated basal and ISO-induced cAMP in these cells. Together, these results suggest that changes to ACV/VI and PDE4 in these cells likely occur due to compensatory mechanisms due to elevated cAMP, caused by a yet to be determined mechanism. Given our results demonstrating variable ROS generation induced by ISO in SAEC versus A-SAEC, we also probed the expression of H_2_O_2_ generating SOD1–3 as well as catalase, which metabolizes H_2_O_2,_ and our results showed no visually significant alterations in their expression, although catalase expression may have been slightly elevated in A-SAEC (Fig. 6F, supplementary figures).

### 3.6. Elevated PDE4 expression in asthma-diseased SAEC contributes to heightened cAMP

**Fig. 7.**
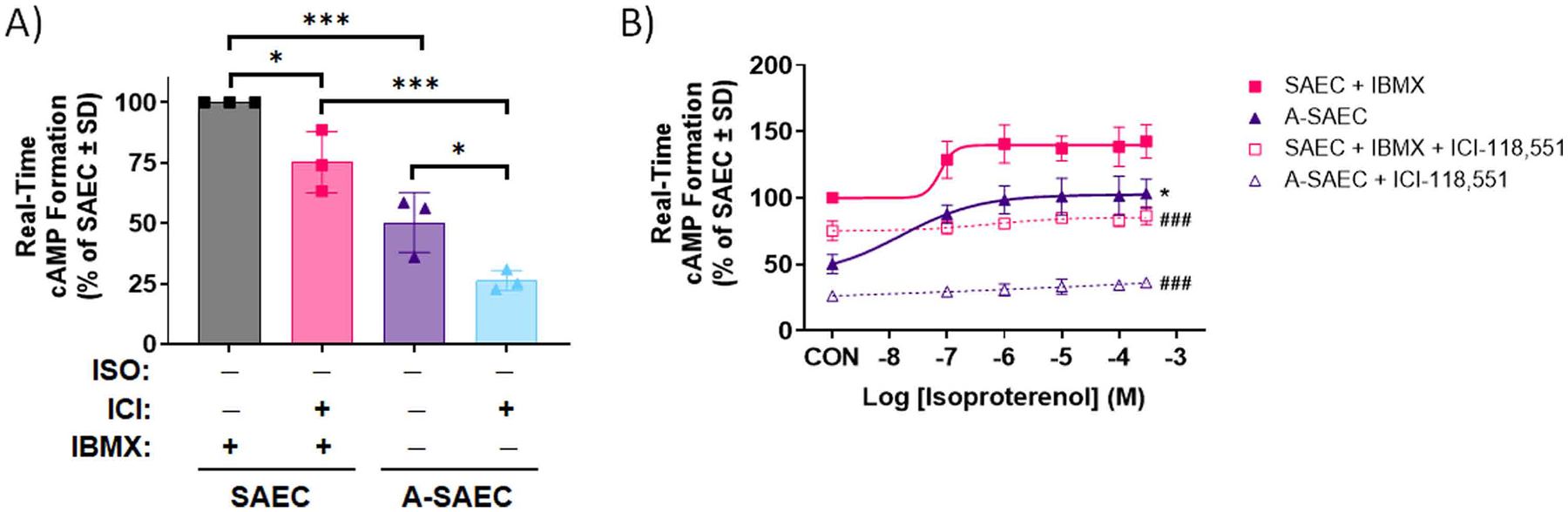
Elevated PDE expression in asthma-SAEC modulates alterations in basal and β2AR-induced cAMP formation. In the absence of IBMX (100μM) basal cAMP response in **(A)** A-SAEC was significantly attenuated (*p* < 0.001) in comparison to healthy SAEC, illustrating the influence of uninhibited and elevated PDE4 activity in decreasing the cAMP signal. The basal cAMP response was abolished in the presence of β_2_AR inverse agonist ICI-118,551 in both SAEC (*p* < 0.05) and A-SAEC (*p* < 0.05). In addition, ISO-induced cAMP formation in SAEC and A-SAEC, with and without IBMX, respectively was completely diminished by ICI-118,551 (100 μM, 5 min) preincubation. Statistical analysis was performed using ANOVA with Tukey’s post-hoc test, and significance is denoted as * *p* < 0.05 or * ** *p* < 0.001, as shown, *n* = 3, with each performed in triplicate. Given the variable concentrations of cAMP in each cell type, data are normalized to a percentage of the SAEC response ± SD. **(B)** The concentration-responses demonstrate decreased cAMP formation in A-SAEC compared to SAEC, with ICI-118,551 flattening the cAMP responses to ISO in both SAEC (*p* < 0.001) and A-SAEC (*p* < 0.001). Statistical analysis was performed using ANOVA with Tukey’s post-hoc test, and significance is denoted as * *p* < 0.05 compared to the SAEC + IBMX condition and ^###^ *p* < 0.001 compared to the respective condition lacking ICI-118,551. Each experiment was independently performed in triplicate twice, and given the variable concentrations of cAMP in each cell type, data are normalized to a percentage of the SAEC response ± SD.

Given the elevated basal and ISO-induced cAMP, and higher expression of PDE4 in asthma-diseased SAEC seen in our studies, we examined the role of PDE inhibition by IBMX used in our assays on the cAMP effect. As noted earlier, cAMP formation assays are typically performed in the presence of the PDE inhibitor IBMX to limit cAMP turnover and make cAMP more robustly quantifiable. However, given our results here, we sought to investigate the role of IBMX in the cAMP formation differences seen here. To gauge this, we performed cAMP formation assays in the presence of IBMX in SAEC, but without the PDE4 inhibitor in A-SAEC, which express higher comparative levels of PDE4. In the absence of ISO, our results demonstrated a large reduction (ca. 50%) in basal cAMP formation in A-SAEC in the absence of IBMX (Fig. 7A). This basal effect was also significantly decreased by ICI-118,551 (Fig. 7A), again demonstrating a role for constitutive β2AR activity in maintenance of basal cAMP levels. On the contrary, healthy SAEC treated with IBMX exhibited significantly higher basal real-time cAMP formation compared to asthma-diseased cells, and this effect was also slightly but significantly reduced by ICI-118,551 (Fig. 7A). The ISO-stimulated real-time cAMP formation was also higher in healthy SAEC with IBMX compared to A-SAEC without IBMX, and the ISO-induced effect was significantly inhibited by ICI-118,551 compared to ISO alone in both SAEC and A-SAEC (Fig. 7B). Together, these results demonstrate that PDE4 overexpression in A-SAEC contributes to the greater real-time cAMP formation seen in these cells, and is due to IBMX-mediated inhibition of PDE in these cells.

## 4. Discussion

We and others have previously described that agonism of β2AR facilitates ROS generation on the order of 1.25–2–fold that of control in a variety of cells and tissues [18–23,25,35,36]. Our previous work using DCFDA-based probes that are non-specific to different ROS species had also noted considerable ROS generation in immortalized CALU-3 cells [18]. In this study, we have shown for the first time that agonism of β2AR specifically generates H_2_O_2_ in A549 and CALU3 airway epithelial cells, as well as small airway epithelial cells from healthy and asthma-diseased human donors. To minimize variability and contamination of other cell types, we utilized commercially available primary epithelial cells from donors, which are validated to contain greater than 90% small airway epithelial cells as assessed by cytokeratin-19 expression. Using a real-time fluorescent assay sensitive to extracellular H_2_O_2_, our results demonstrate elevations of ISO-induced H_2_O_2_ in SAEC at concentrations above 10 nM. Interestingly, ISO-induced H_2_O_2_ generation was sensitive to the NOX inhibitor VAS2870 in CALU-3 cells and healthy SAEC, but not in asthma-diseased SAEC when using the extracellular H_2_O_2_ probe. Of note, VAS2870 alone did significantly decrease H_2_O_2_ generation in both SAEC types. This difference in the presence of agonist could potentially be due to distinct sources of H_2_O_2_, for example mitochondrial ROS generation, in the asthma disease state. However, the ROS generating effects of ISO as detected by the intracellular H_2_O_2_ probe were sensitive to the NOX inhibitor in both healthy and diseased SAEC, making it more likely that there are potential differences in diffusability or production of extracellular H_2_O_2_ in the asthma SAEC. Consistent with this, extracellular superoxide dismutase (SOD3, ecSOD), which rapidly generates extracellular H_2_O_2_ from superoxide is upregulated in asthmatic patients [62], yet interestingly, our data did not reveal significant alterations in SOD3 expression in the A-SAEC cells used here versus the SAEC, although catalase expression was slightly visually, but not significantly, elevated in the A-SAEC cells. Importantly, our results also affirm the role of β2AR in H_2_O_2_ generation, as we and others have noted previously, the selective β2-inverse agonist fully blocked ISO-induced H_2_O_2_ generation, similar to the effects of VAS2870 in both SAEC types. Together, these results demonstrate that β2AR agonism can specifically generate H_2_O_2_ in a receptor and NOX-dependent manner in SAEC.

While we have previously demonstrated that H_2_O_2_ and β2AR agonism can facilitate transient cysteine-S-sulfenation of the β2AR, a novel and principal finding of our current study is that acute treatment of healthy or asthma-diseased SAEC with the fully efficacious β-agonist ISO or the clinically used and selective β2-partial agonist salbutamol induces formation of higher-order cysteine-S-sulfinic acids of β2AR, as detected by a clickable, selective Cys-S-sulfinic acid probe. Moreover, twice daily administration of ISO or H_2_O_2_ for seven days similarly induced elevated levels of S-sulfinated β2AR. Our previous results showed that oxidation of the β2AR to the cysteine-S-sulfenic acid (SOH) oxidized state enhances β2AR function, but that entrapment of the cysteine-S-sulfenic acid with the S-sulfenic acid probe dimedone, which mimics irreversible S-sulfination and prevents normal redox cycling, reduces β2AR signaling, including cAMP formation [18]. Our current results are consistent with this and demonstrate that lower levels of H_2_O_2_ enhance ISO-induced cAMP formation, while high oxidant levels (1 mM H_2_O_2_) inhibit β2AR-mediated cAMP formation, and that this effect can be rescued in the presence of the H_2_O_2_ scavenger and cysteine-donor NAC. Notably, 1 mM H_2_O_2_ also decreased basal cAMP formation, however, our results with ICI-118,551 demonstrate that constitutive β2AR activity plays a significant role in regulating this basal cAMP in both types of SAEC. Together, these results suggest that hyperoxidation of β2AR to Cys-S-sulfinic acids may impair β2AR function and contribute to tachyphylaxis responses seen upon clinical use of β2-agonists. This could be especially important given that β2AR agonism itself generates ROS in the airway epithelium, and also since the asthma-disease state is characterized by heightened level of ROS, including NOX4 upregulation in both airway epithelial cells and the underlying smooth muscle [5, 10–13]. Together, these effects could likely contribute to increased hyperoxidation of β2AR to the S-sulfinic acid form, which is irreversible and functionally impaired, and as such may be an additional mechanism that explains tachyphylaxis to β2-agonists seen in asthmatic patients. Further studies are underway in our laboratory to extend this work in vivo and also gauge the effects of epithelial-generated H_2_O_2_ on β2AR localized on the underlying smooth muscle cells that drive the heightened airway tone.

A surprising finding of the current study was the significantly higher levels of both basal and ISO-induced cAMP formation in small airway epithelial cells from the asthma-diseased patient compared to the healthy SAEC. This elevation was not due to higher levels of β2AR, Gαs or ACV/VI, and in fact, our data revealed a subtle but consistent (amongst *n* = 4) decrease in ACV/VI in A-SAEC compared to healthy SAEC, which is contradictory to the levels of cAMP seen in A-SAEC compared to SAEC. Moreover, Gαi1/2 expression was not significantly altered in between the cell types suggesting that the well-described Gαs/Gαi-switch is not responsible for the noted differences in cAMP levels. Interestingly, we did note a significantly higher level of PDE4, a principal phosphodiesterase isoform expressed in the airway epithelium [57–61], in the SAEC derived from the asthmatic patient compared to healthy SAEC. While this noted increase in PDE4 expression is evident, we cannot discount the possibility that higher levels of ROS alter PDE4 activity, necessitating upregulation of its expression. Nonetheless, both the decreased AC and increased PDE would be expected to significantly decrease cAMP concentrations in A-SAEC and as a consequence, we hypothesize that these effects are indeed likely compensatory to the heightened cAMP in these cells. While IBMX plays a key role in inhibition of PDE and resulting cAMP levels, the reasons for these altered levels of expression remain unclear. Similarly, while we have used single-patient derived SAEC to decrease confounding variables like patient age, pharmacological treatments, and comorbidities, a limitation of our results is that the data are reflective of a homogenous cell population. Nonetheless, these results represent only the initial investigations to our knowledge on the role of the β2AR-ROS signaling axis in primary airway epithelial cells. Further work will need to be performed in a more heterogenous sampling of asthma-diseased cells to determine the significance and mechanisms of this increase in cAMP in these cells.

In the present report, we demonstrate that agonism of β2AR endogenously expressed on the surface of airway epithelial cells results in generation of H_2_O_2_, and this effect as well as exogenous H_2_O_2_ can induce cysteine-S-sulfination of the receptor, which has deleterious effects on receptor function, and can be rescued, at least in part, by cysteine-donating antioxidants. These results may have implications towards explaining the tachyphylaxis towards β2-agonists seen clinically. Further research is underway in our laboratory to assess the in vivo effects of β2-agonists on this phenomenon.

## 5. Conclusions

Our results demonstrate that β2AR agonism can generate H_2_O_2_ in airway epithelial cells, which together with the already heightened oxidant burden in the asthmatic airway epithelia can overoxidize the β2AR forming dysfunctional β2AR cysteine-S-sulfinic acids. These results suggest a model whereby hyperoxidized β2AR may explain the loss of β2-agonist efficacy seen in clinical tachyphylaxis.

## Supplementary Material

Supplementary File

## List of abbreviations:

AC: adenylyl cyclase
A-SAEC: asthmatic small airway epithelial cells
β2AR: β2-adrenergic receptors
cAMP: adenosine 3’, 5’-cyclic monophosphate
H_2_O_2_: hydrogen peroxide
PDE: phosphodiesterase
ROS: reactive oxygen species
SAEC: small airway epithelial cells
SOH: S-sulfenic acid
SO_2_H: S-sulfinic acid

## References

[1] Cooper PR, Panettieri RA, Steroids completely reverse albuterol-induced β2-adrenergic receptor tolerance in human small airways, J. Allergy Clin. Immunol 122 (4) (2008) 734–740. 18774166 10.1016/j.jaci.2008.07.040

[2] Hancox RJ, Subbarao P, Kamada D, Watson RM, Hargreave FE, Inman MD, β2-agonist tolerance and exercise-induced bronchospasm, Am. J. Respir. Crit. Care Med 165 (8) (2002) 1068–1070. 11956046 10.1164/ajrccm.165.8.200111-091bc

[3] Yim RP, Koumbourlis AC, Tolerance & resistance to β2-agonist bronchodilators. Paediatr. Respir. Rev, W.B. Saunders, 2013, pp. 195–198. 10.1016/j.prrv.2012.11.002 23507501

[4] Heijink IH, Kuchibhotla VNS, Roffel MP, Maes T, Knight DA, Sayers I, Nawijn MC, Epithelial cell dysfunction, a major driver of asthma development. Allergy: European Journal of Allergy and Clinical Immunology, Blackwell Publishing Ltd, 2020, pp. 1898–1913. 10.1111/all.14421 PMC7496351 32460363

[5] Potaczek DP, Miethe S, Schindler V, Alhamdan F, Garn H, Role of airway epithelial cells in the development of different asthma phenotypes, Cell. Signal 69 (2020), 109523. 31904412 10.1016/j.cellsig.2019.109523

[6] Pŕefontaine D, Hamid Q, Airway epithelial cells in asthma, J. Allergy Clin. Immunol 120 (6) (2007) 1475–1478. 17980414 10.1016/j.jaci.2007.09.041

[7] Sahiner UM, Birben E, Erzurum S, Sackesen C, Kalayci O, Oxidative stress in asthma. World Allergy Organization Journal, BioMed Central Ltd, 2011, pp. 151–158. 23268432 10.1097/WOX.0b013e318232389e PMC3488912

[8] Sugiura H, Ichinose M, Oxidative and nitrative stress in bronchial asthma, Antioxid. Redox Signal., Antioxid. Redox Signal (2008) 785–797. 18177234 10.1089/ars.2007.1937

[9] Wang YL, Bai C, Li K, Adler KB, Wang X, Role of airway epithelial cells in development of asthma and allergic rhinitis, Respir. Med, W. B. Saunders (2008) 949–955. 18339528 10.1016/j.rmed.2008.01.017

[10] Albano GD, Gagliardo RP, Montalbano AM, Profita M, Overview of the mechanisms of oxidative stress: impact in inflammation of the airway diseases, Antioxidants 11 (11) (2022). 10.3390/antiox11112237 PMC9687037 36421423

[11] Michaeloudes C, Abubakar-Waziri H, Lakhdar R, Raby K, Dixey P, Adcock IM, Mumby S, Bhavsar PK, Chung KF, Molecular mechanisms of oxidative stress in asthma, Mol. Asp. Med 85 (2022), 101026. 10.1016/j.mam.2021.101026 34625291

[12] Hollins F, Sutcliffe A, Gomez E, Berair R, Russell R, Szyndralewiez C, Saunders R, Brightling C, Airway smooth muscle NOX4 is upregulated and modulates ROS generation in COPD, Respir. Res 17 (1) (2016), 84. 27435477 10.1186/s12931-016-0403-y PMC4950777

[13] Wan WY, Hollins F, Haste L, Woodman L, Hirst RA, Bolton S, Gomez E, Sutcliffe A, Desai D, Chachi L, Mistry V, Szyndralewiez C, Wardlaw A, Saunders R, O’Callaghan C, Andrew PW, Brightling CE, NADPH oxidase-4 overexpression is associated with epithelial ciliary dysfunction in neutrophilic asthma, Chest 149 (6) (2016) 1445–1459. 26836936 10.1016/j.chest.2016.01.024 PMC4893823

[14] Nisimoto Y, Diebold BA, Cosentino-Gomes D, Lambeth JD, Nox4: a hydrogen peroxide-generating oxygen sensor, Biochemistry 53 (31) (2014) 5111–5120. 25062272 10.1021/bi500331y PMC4131900

[15] Serrander L, Cartier L, Bedard K, Banfi B, Lardy B, Plastre O, Sienkiewicz A, Fórró L, Schlegel W, Krause K-H, NOX4 activity is determined by mRNA levels and reveals a unique pattern of ROS generation, Biochem. J 406 (1) (2007) 105–114. 17501721 10.1042/BJ20061903 PMC1948990

[16] Takac I, Schröder K, Zhang L, Lardy B, Anilkumar N, Lambeth JD, Shah AM, Morel F, Brandes RP, The E-loop is involved in hydrogen peroxide formation by the NADPH oxidase Nox4, J. Biol. Chem 286 (15) (2011) 13304–13313. 21343298 10.1074/jbc.M110.192138 PMC3075677

[17] Rambacher KM, Moniri NH, The β2-adrenergic receptor-ROS signaling axis: an overlooked component of β2AR function? Biochem. Pharmacol (2020). 10.1016/j.bcp.2019.113690 PMC6917825 31697929

[18] Rambacher KM, Moniri NH, Cysteine redox state regulates human β2-adrenergic receptor binding and function, Sci. Rep 10 (1) (2020), 2934–2934. 32076070 10.1038/s41598-020-59983-4 PMC7031529

[19] Singh M, Moniri NH, Reactive oxygen species are required for β2 adrenergic receptor-β-arrestin interactions and signaling to ERK1/2, Biochem. Pharmacol 84 (5) (2012) 661–669. 22728070 10.1016/j.bcp.2012.06.012

[20] Moniri NH, Daaka Y, Agonist-stimulated reactive oxygen species formation regulates beta2-adrenergic receptor signal transduction, Biochem. Pharmacol 74 (1) (2007) 64–73. 17451656 10.1016/j.bcp.2007.03.016

[21] Kondo H, Takeuchi S, Togari A, β-Adrenergic signaling stimulates osteoclastogenesis via reactive oxygen species, Am. J. Physiol. Endocrinol. Metab 304 (5) (2013) E507–E515. 23169789 10.1152/ajpendo.00191.2012

[22] Davel AP, Brum PC, Rossoni LV, Isoproterenol induces vascular oxidative stress and endothelial dysfunction via a Giα-coupled β2-adrenoceptor signaling pathway, PLoS One 9 (3) (2014), e91877. 24622771 10.1371/journal.pone.0091877 PMC3951496

[23] Qian L, Hu X, Zhang D, Snyder A, Wu HM, Li Y, Wilson B, Lu RB, Hong JS, Flood PM, beta2 Adrenergic receptor activation induces microglial NADPH oxidase activation and dopaminergic neurotoxicity through an ERK-dependent/protein kinase A-independent pathway, Glia 57 (15) (2009) 1600–1609. 19330844 10.1002/glia.20873 PMC3608678

[24] Bovo E, Lipsius SL, Zima AV, Reactive oxygen species contribute to the development of arrhythmogenic Ca^2^+ waves during β-adrenergic receptor stimulation in rabbit cardiomyocytes, J. Physiol 590 (14) (2012) 3291–3304. 22586224 10.1113/jphysiol.2012.230748 PMC3459043

[25] Li J, Yan B, Huo Z, Liu Y, Xu J, Sun Y, Liu Y, Liang D, Peng L, Zhang Y, Zhou ZN, Shi J, Cui J, Chen YH, beta2- but not beta1-adrenoceptor activation modulates intracellular oxygen availability, J. Physiol 588 (Pt 16) (2010) 2987–2998. 20547682 10.1113/jphysiol.2010.190900 PMC2956940

[26] Chiarella SE, Soberanes S, Urich D, Morales-Nebreda L, Nigdelioglu R, Green D, Young JB, Gonzalez A, Rosario C, Misharin AV, Ghio AJ, Wunderink RG, Donnelly HK, Radigan KA, Perlman H, Chandel NS, Budinger GRS, Mutlu GM, β_2_-Adrenergic agonists augment air pollution-induced IL-6 release and thrombosis, J. Clin. Investig 124 (7) (2014) 2935–2946. 24865431 10.1172/JCI75157 PMC4071386

[27] Gong K, Li Z, Xu M, Du J, Lv Z, Zhang Y, A novel protein kinase A-independent, beta-arrestin-1-dependent signaling pathway for p38 mitogen-activated protein kinase activation by beta2-adrenergic receptors, J. Biol. Chem 283 (43) (2008) 29028–29036. 18678875 10.1074/jbc.M801313200 PMC2662007

[28] Burns RN, Moniri NH, Agonist- and hydrogen peroxide-mediated oxidation of the β2 adrenergic receptor: evidence of receptor S-sulfenation as detected by a modified biotin-switch assay, J. Pharmacol. Exp. Ther 339 (3) (2011) 914–921. 21917560 10.1124/jpet.111.185975

[29] Leonard SE, Reddie KG, Carroll KS, Mining the thiol proteome for sulfenic acid modifications reveals new targets for oxidation in cells, ACS Chem. Biol 4 (9) (2009) 783–799. 10.1021/cb900105q 19645509

[30] Reddie KG, Carroll KS, Expanding the functional diversity of proteins through cysteine oxidation, Curr. Opin. Chem. Biol 12 (6) (2008) 746–754. 18804173 10.1016/j.cbpa.2008.07.028

[31] Gupta V, Carroll KS, Sulfenic acid chemistry, detection and cellular lifetime, Biochim. Biophys. Acta 1840 (2) (2014) 847–875. 23748139 10.1016/j.bbagen.2013.05.040 PMC4184475

[32] Paulsen CE, Carroll KS, Orchestrating redox signaling networks through regulatory cysteine switches, ACS Chem. Biol 5 (1) (2010) 47–62. 19957967 10.1021/cb900258z PMC4537063

[33] Cheshmehkani A, Senatorov IS, Dhuguru J, Ghoneim O, Moniri NH, Free-fatty acid receptor-4 (FFA4) modulates ROS generation and COX-2 expression via the C-terminal β-arrestin phosphosensor in Raw 264.7 macrophages, Biochem. Pharmacol (2017). 10.1016/j.bcp.2017.09.008 PMC5705417 28943238

[34] Senatorov IS, Cheshmehkani A, Burns RN, Singh K, Moniri NH, Carboxy-terminal phosphoregulation of the long splice isoform of free-fatty acid receptor-4 mediates β -arrestin recruitment and signaling to ERK1/2, Mol. Pharmacol 97 (5) (2020) 304–313. 32132133 10.1124/mol.119.117697 PMC7081053

[35] Bovo E, Lipsius SL, Zima AV, Reactive oxygen species contribute to the development of arrhythmogenic Ca^2^+ waves during β-adrenergic receptor stimulation in rabbit cardiomyocytes, J. Physiol 590 (14) (2012) 3291–3304. 22586224 10.1113/jphysiol.2012.230748 PMC3459043

[36] Chiarella SE, Soberanes S, Urich D, Morales-Nebreda L, Nigdelioglu R, Green D, Young JB, Gonzalez A, Rosario C, Misharin AV, Ghio AJ, Wunderink RG, Donnelly HK, Radigan KA, Perlman H, Chandel NS, Budinger GR, Mutlu GM, β_2_-Adrenergic agonists augment air pollution-induced IL-6 release and thrombosis, J. Clin. Invest 124 (7) (2014) 2935–2946. 24865431 10.1172/JCI75157 PMC4071386

[37] Carstairs JR, Nimmo AJ, Barnes PJ, Autoradiographic visualization of beta-adrenoceptor subtypes in human lung, Am. Rev. Respir. Dis 132 (3) (1985) 541–547. 2864008 10.1164/arrd.1985.132.3.541

[38] Mak JC, Grandordy B, Barnes PJ, High affinity [3H]formoterol binding sites in lung: characterization and autoradiographic mapping, Eur. J. Pharmacol 269 (1) (1994) 35–41. 7828656 10.1016/0922-4106(94)90023-x

[39] Singh M, Moniri NH, Reactive oxygen species as β2-adrenergic receptor signal transducers, J. Pharm. Pharmacol 2 (1) (2014).

[40] Qian L, Hu X, Zhang D, Snyder A, Wu H-M, Li Y, Wilson B, Lu R-B, Hong JS, Flood PM, beta2 Adrenergic receptor activation induces microglial NADPH oxidase activation and dopaminergic neurotoxicity through an ERK-dependent/protein kinase A-independent pathway, Glia 57 (15) (2009) 1600–1609. 19330844 10.1002/glia.20873 PMC3608678

[41] Akter S, Fu L, Jung Y, Conte ML, Lawson JR, Lowther WT, Sun R, Liu K, Yang J, Carroll KS, Chemical proteomics reveals new targets of cysteine sulfinic acid reductase, Nat. Chem. Biol 14 (11) (2018) 995–1004. 30177848 10.1038/s41589-018-0116-2 PMC6192846

[42] Lo Conte M, Lin J, Wilson MA, Carroll KS, A chemical approach for the detection of protein sulfinylation, ACS Chem. Biol 10 (8) (2015) 1825–1830. 26039147 10.1021/acschembio.5b00124 PMC4605140

[43] Meng J, Fu L, Liu K, Tian C, Wu Z, Jung Y, Ferreira RB, Carroll KS, Blackwell TK, Yang J, Global profiling of distinct cysteine redox forms reveals wide-ranging redox regulation in C. elegans, Nat. Commun 12 (1) (2021), 1415. 33658510 10.1038/s41467-021-21686-3 PMC7930113

[44] Shi Y, Carroll KS, Activity-based sensing for site-specific proteomic analysis of cysteine oxidation, Acc. Chem. Res 53 (1) (2020) 20–31. 31869209 10.1021/acs.accounts.9b00562 PMC7061859

[45] Dougall IG, Harper D, Jackson DM, Leff P, Estimation of the efficacy and affinity of the beta 2-adrenoceptor agonist salmeterol in guinea-pig trachea, Br. J. Pharmacol 104 (4) (1991) 1057–1061. 1687365 10.1111/j.1476-5381.1991.tb12549.x PMC1908838

[46] van der Westhuizen ET, Breton B, Christopoulos A, Bouvier M, Quantification of ligand bias for clinically relevant β2-adrenergic receptor ligands: implications for drug taxonomy, Mol. Pharmacol 85 (3) (2014) 492–509. 24366668 10.1124/mol.113.088880

[47] Kume H, Clinical Use of β2-adrenergic receptor agonists based on their intrinsic efficacy, Allergol. Int 54 (1) (2005) 89–97.

[48] Valko M, Leibfritz D, Moncol J, Cronin MT, Mazur M, Telser J, Free radicals and antioxidants in normal physiological functions and human disease, Int. J. Biochem. Cell Biol 39 (1) (2007) 44–84. 16978905 10.1016/j.biocel.2006.07.001

[49] Davies MJ, Protein oxidation and peroxidation, Biochem. J 473 (7) (2016) 805–825. 27026395 10.1042/BJ20151227 PMC4819570

[50] Lee YM, He W, Liou YC, The redox language in neurodegenerative diseases: oxidative post-translational modifications by hydrogen peroxide, Cell Death Dis. 12 (1) (2021) 58. 33431811 10.1038/s41419-020-03355-3 PMC7801447

[51] Lennicke C, Cochemé HM, Redox metabolism: ROS as specific molecular regulators of cell signaling and function, Mol. Cell 81 (18) (2021) 3691–3707. 34547234 10.1016/j.molcel.2021.08.018

[52] Sies H, Jones DP, Reactive oxygen species (ROS) as pleiotropic physiological signalling agents, Nat. Rev. Mol. Cell Biol 21 (7) (2020) 363–383. 32231263 10.1038/s41580-020-0230-3

[53] Spadaro D, Yun BW, Spoel SH, Chu C, Wang YQ, Loake GJ, The redox switch: dynamic regulation of protein function by cysteine modifications, Physiol. Plant 138 (4) (2010) 360–371. 19912563 10.1111/j.1399-3054.2009.01307.x

[54] Birrell MA, Bonvini SJ, Wortley MA, Buckley J, Yew-Booth L, Maher SA, Dale N, Dubuis ED, Belvisi MG, The role of adenylyl cyclase isoform 6 in β-adrenoceptor signalling in murine airways, Br. J. Pharmacol 172 (1) (2015) 131–141. 25205328 10.1111/bph.12905 PMC4280973

[55] Agarwal SR, Fiore C, Miyashiro K, Ostrom RS, Harvey RD, Effect of adenylyl cyclase type 6 on localized production of cAMP by β−2 adrenoceptors in human airway smooth-muscle cells, J. Pharmacol. Exp. Ther 370 (1) (2019) 104–110. 31068382 10.1124/jpet.119.256594 PMC6548981

[56] Daaka Y, Luttrell LM, Lefkowitz RJ, Switching of the coupling of the beta2-adrenergic receptor to different G proteins by protein kinase A, Nature 390 (6655) (1997) 88–91. 9363896 10.1038/36362

[57] Barnes AP, Livera G, Huang P, Sun C, O’Neal WK, Conti M, Stutts MJ, Milgram SL, Phosphodiesterase 4D forms a cAMP diffusion barrier at the apical membrane of the airway epithelium, J. Biol. Chem 280 (9) (2005) 7997–8003. 15611099 10.1074/jbc.M407521200

[58] BinMahfouz H, Borthakur B, Yan D, George T, Giembycz MA, Newton R, Superiority of combined phosphodiesterase PDE3/PDE4 inhibition over PDE4 inhibition alone on glucocorticoid- and long-acting β2-adrenoceptor agonist-induced gene expression in human airway epithelial cells, Mol. Pharmacol 87 (1) (2015) 64–76. 25324049 10.1124/mol.114.093393

[59] Blanchard E, Zlock L, Lao A, Mika D, Namkung W, Xie M, Scheitrum C, Gruenert DC, Verkman AS, Finkbeiner WE, Conti M, Richter W, Anchored PDE4 regulates chloride conductance in wild-type and ΔF508-CFTR human airway epithelia, FASEB J. 28 (2) (2014) 791–801. 24200884 10.1096/fj.13-240861 PMC3898646

[60] Fuhrmann M, Jahn HU, Seybold J, Neurohr C, Barnes PJ, Hippenstiel S, Kraemer HJ, Suttorp N, Identification and function of cyclic nucleotide phosphodiesterase isoenzymes in airway epithelial cells, Am. J. Respir. Cell Mol. Biol 20 (2) (1999) 292–302. 9922221 10.1165/ajrcmb.20.2.3140

[61] Zuo H, Han B, Poppinga WJ, Ringnalda L, Kistemaker LEM, Halayko AJ, Gosens R, Nikolaev VO, Schmidt M, Cigarette smoke up-regulates PDE3 and PDE4 to decrease cAMP in airway cells, Br. J. Pharmacol 175 (14) (2018) 2988–3006. 29722436 10.1111/bph.14347 PMC6016635

[62] Gaurav R, Varasteh JT, Weaver MR, Jacobson SR, Hernandez-Lagunas L, Liu Q, Nozik-Grayck E, Chu HW, Alam R, Nordestgaard BG, Kobylecki CJ, Afzal S, Chupp GL, Bowler RP, The R213G polymorphism in SOD3 protects against allergic airway inflammation, JCI Insight 2 (17) (2017). 10.1172/jci.insight.95072 PMC5621928 28878123

