# Supplementary material for: Agonists and hydrogen peroxide mediate hyperoxidation of β2-adrenergic receptor in airway epithelial cells: Implications for tachyphylaxis to β2-agonists in constrictive airway disorders
Source: Biomed Pharmacother. Author manuscript; Available in PMC 2024 Feb 5. (PMC10842251; doi:10.1016/j.biopha.2023.115763)

Supplementary Figures

Singh K and Moniri NH. β2-adrenergic receptor-reactive oxygen species signaling axis in small airway epithelial cells: agonist and H2O2-mediated hyperoxidation of β2AR and implications for tachyphylaxis to β2-agonists.

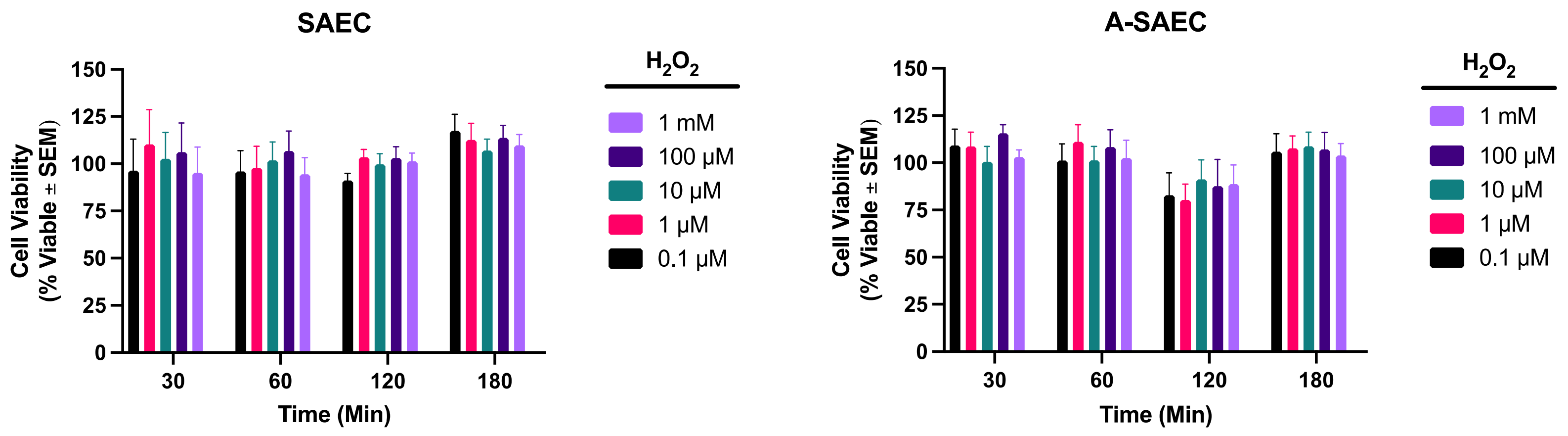

Supplementary Figure 1: Cell viability assays of SAEC and A-SAEC in the presence of increasing concentrations of H_2O_2 for 30-180 min yields no significant differences in cell viability.

Full length immunoblot replicates from figure 2A:

SAEC blots:

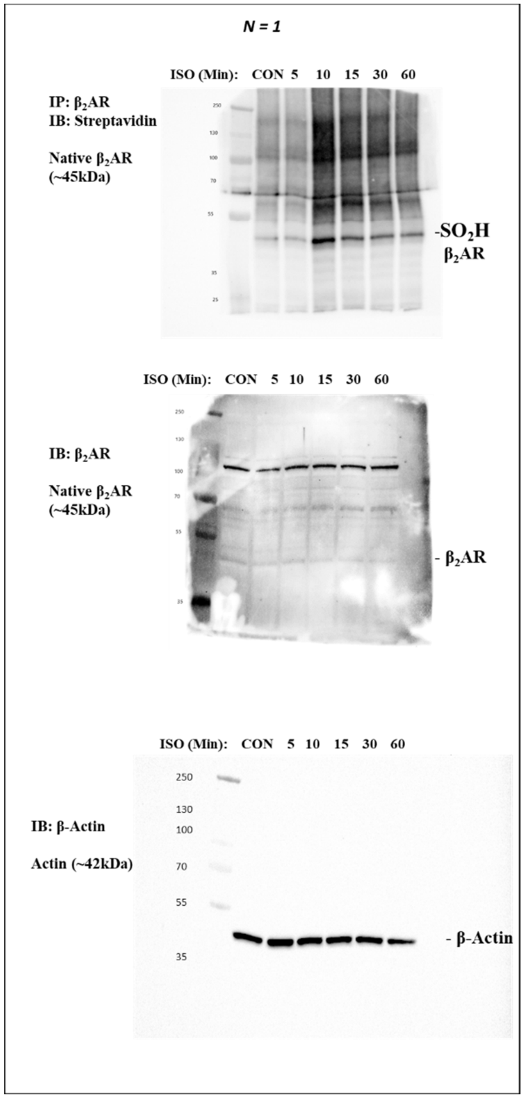

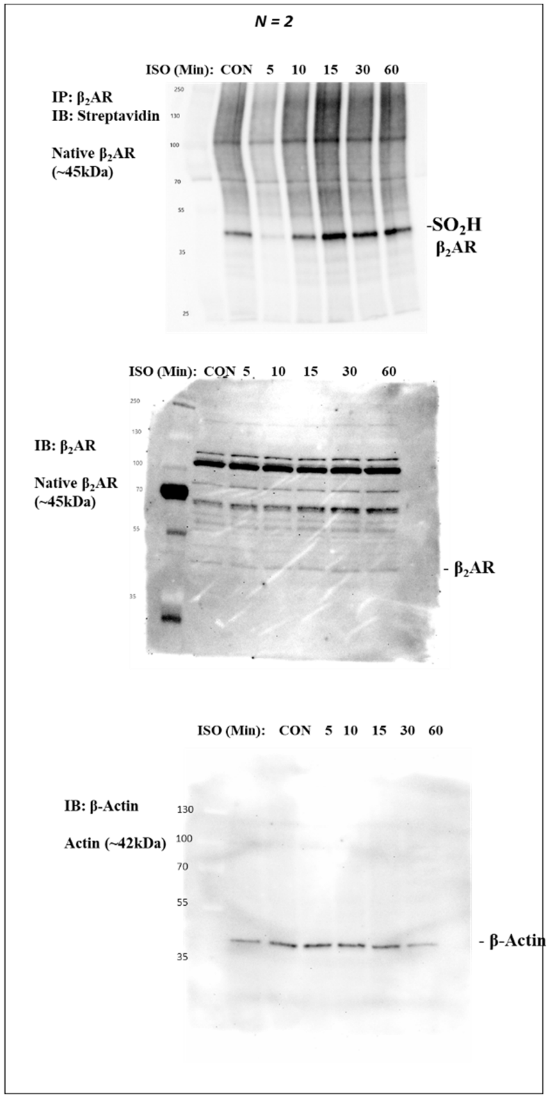

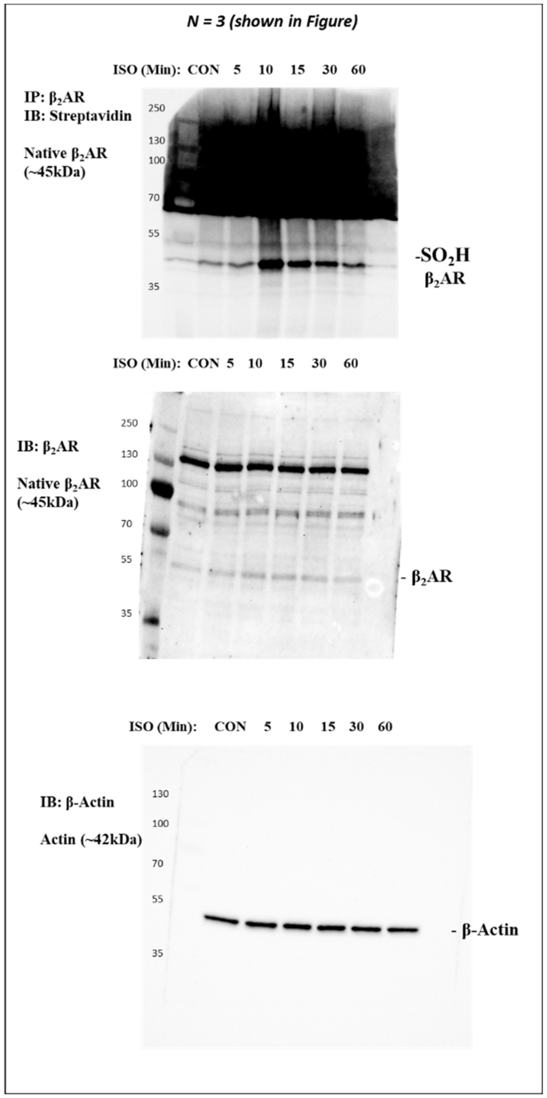

Full length immunoblot replicates from figure 2A:

A-SAEC blots:

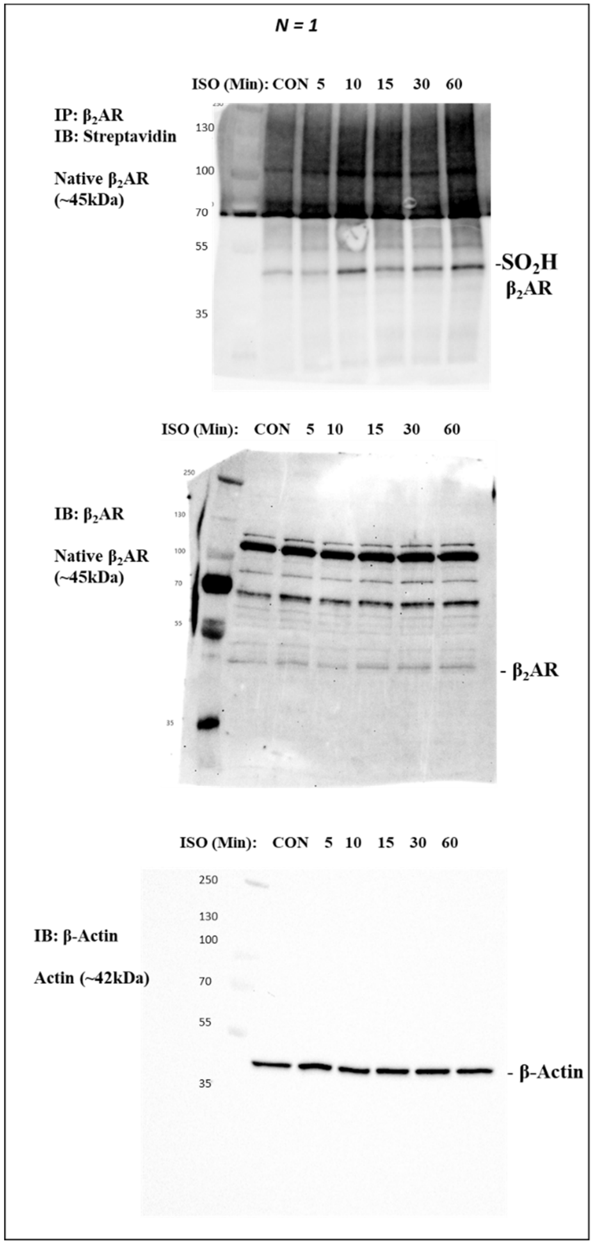

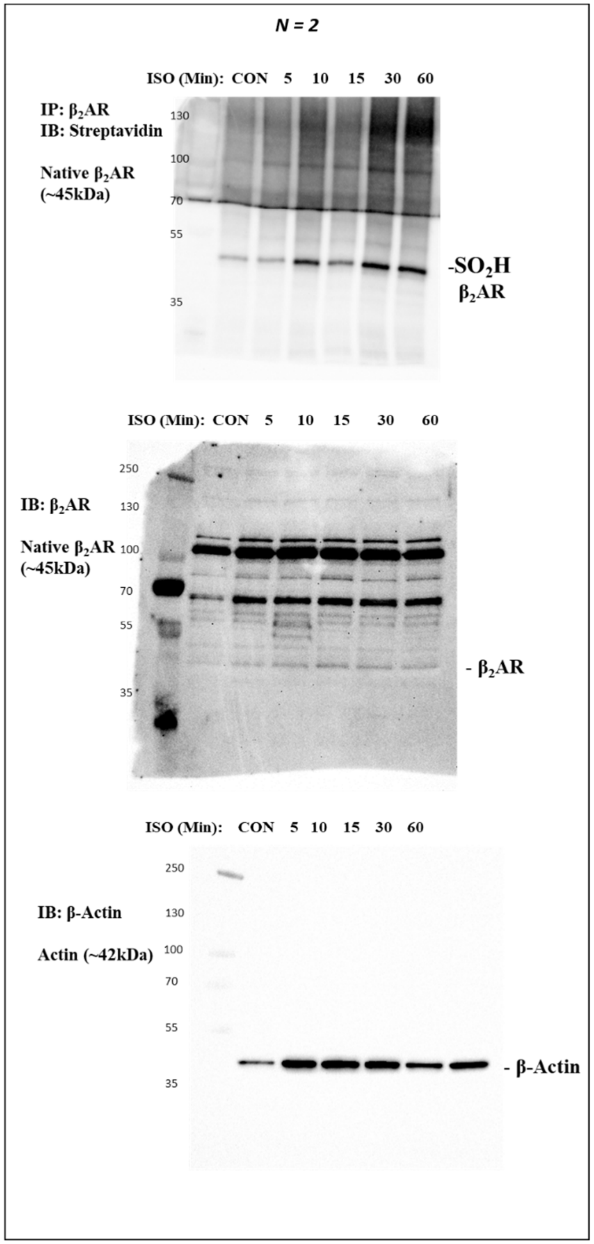

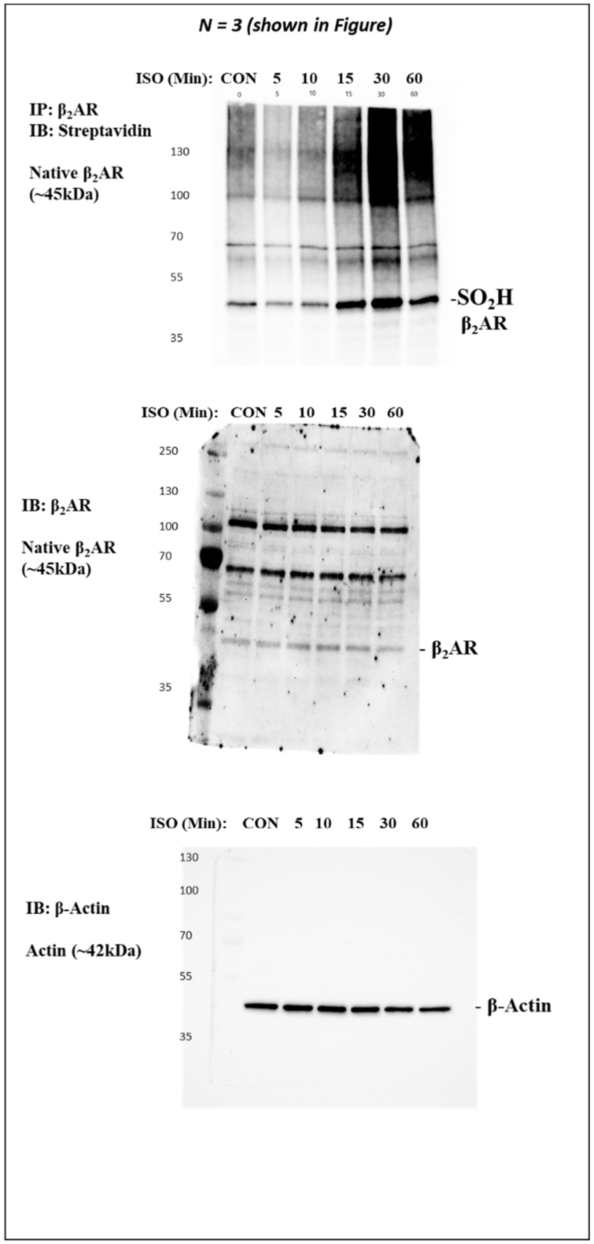

Full length immunoblot replicates from figure 2B:

SAEC blots:

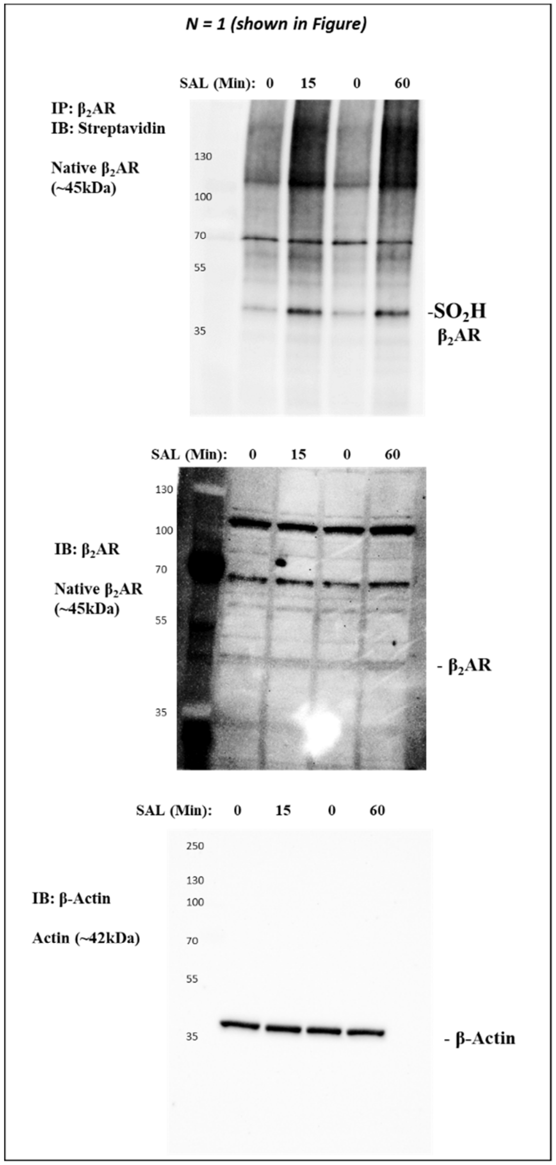

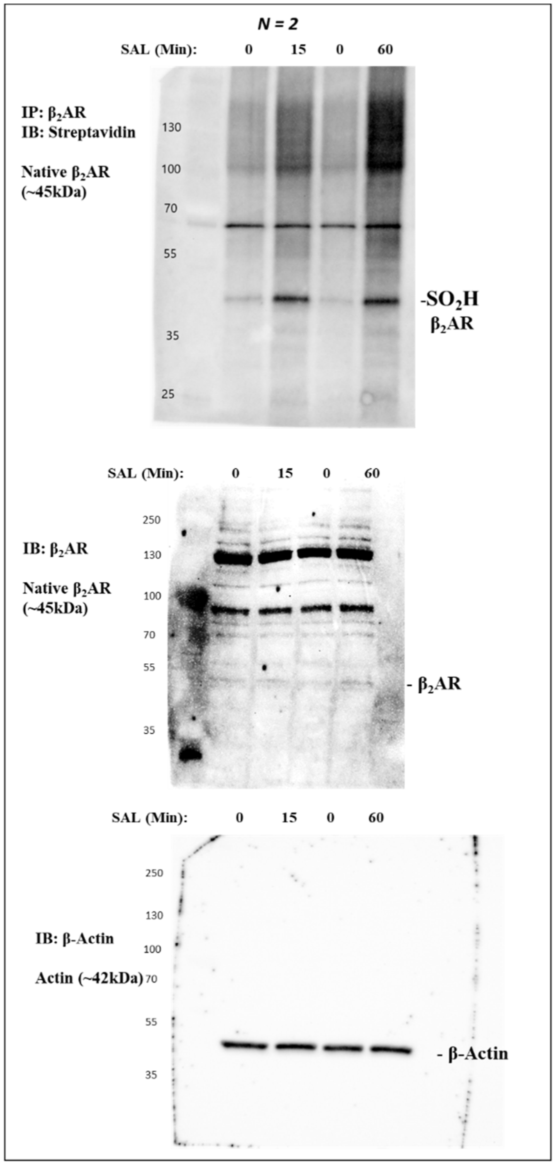

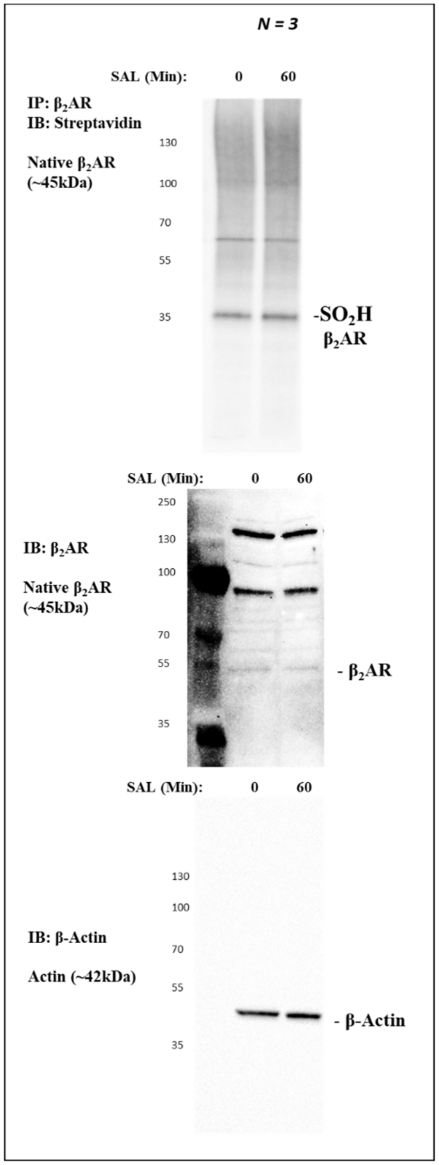

Full length immunoblot replicates from figure 2B:

A-SAEC blots:

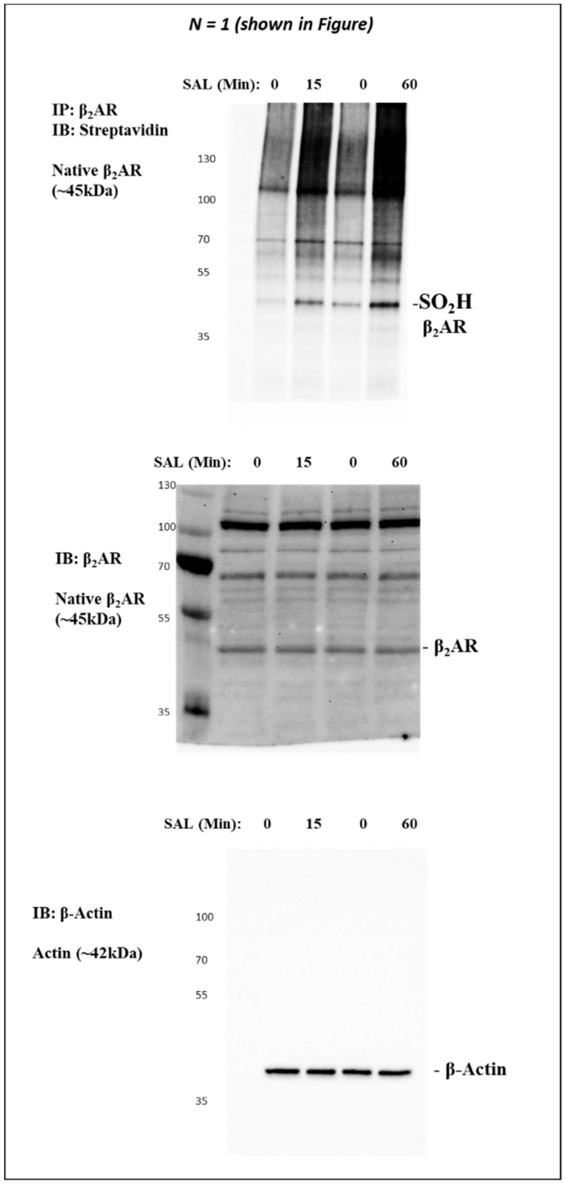

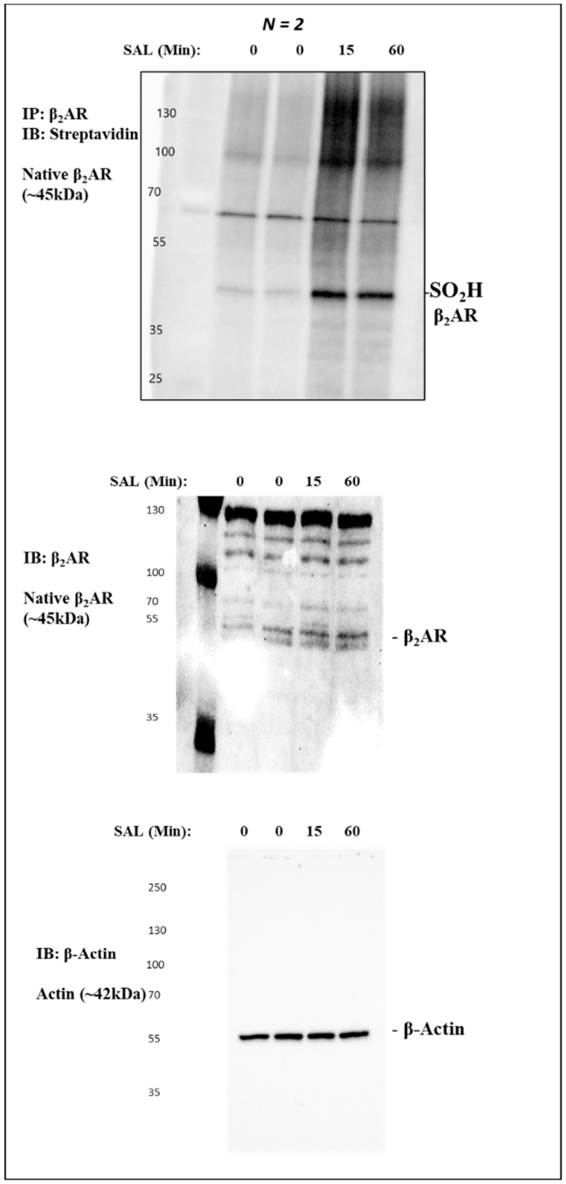

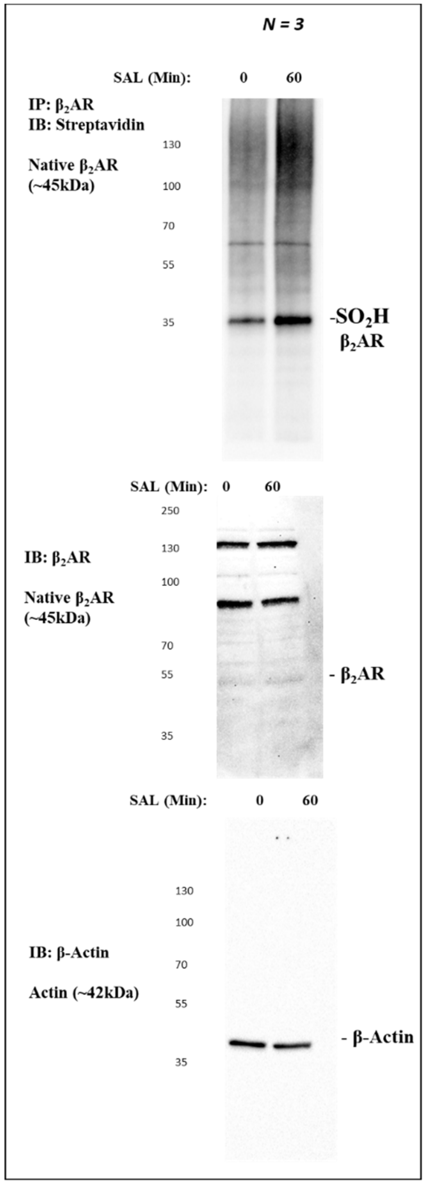

Full length immunoblot replicates from figure 2C:

SAEC – ISO blots

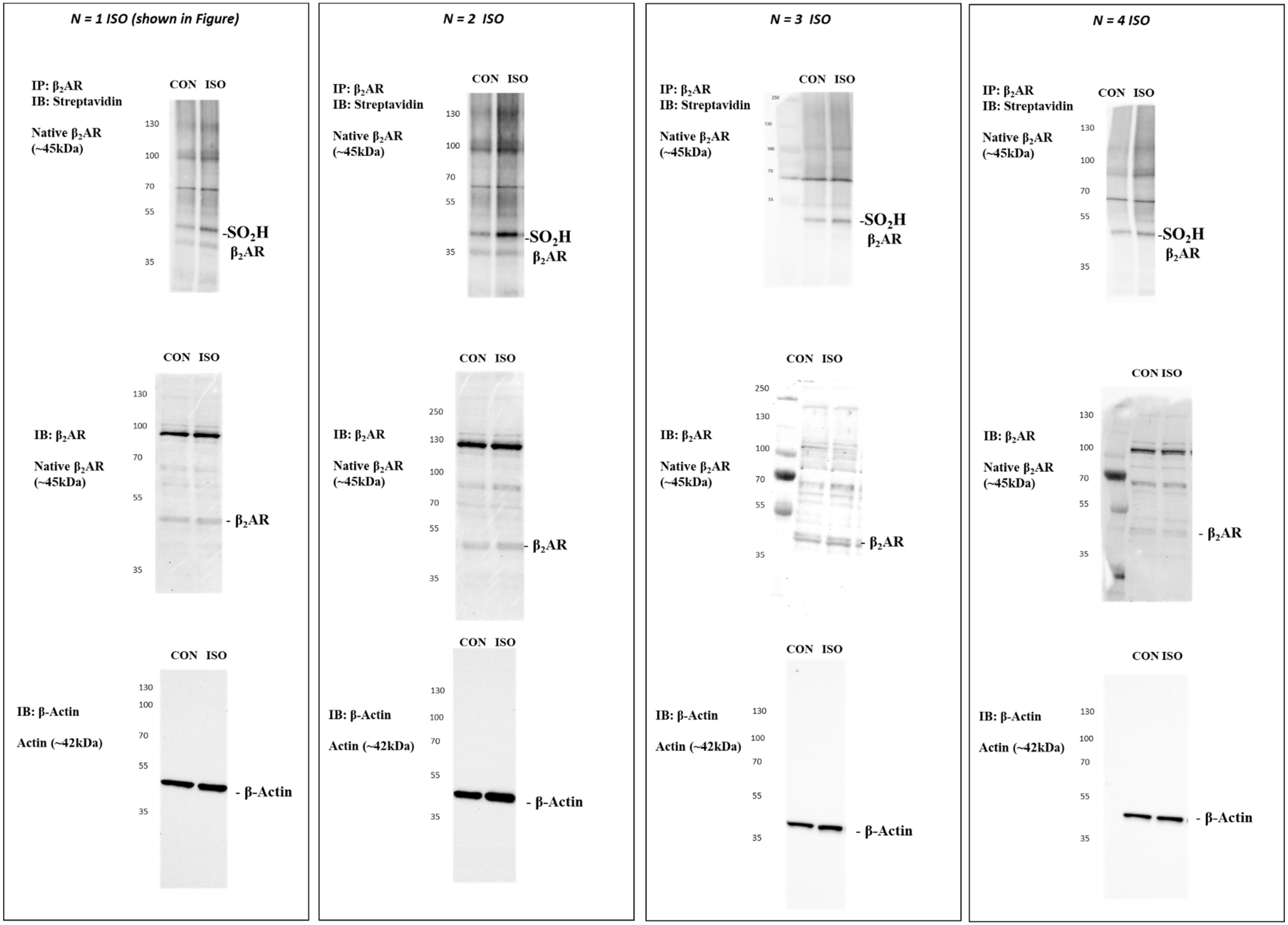

Full length immunoblot replicates from figure 2C:

SAEC – H_2O_2 blots

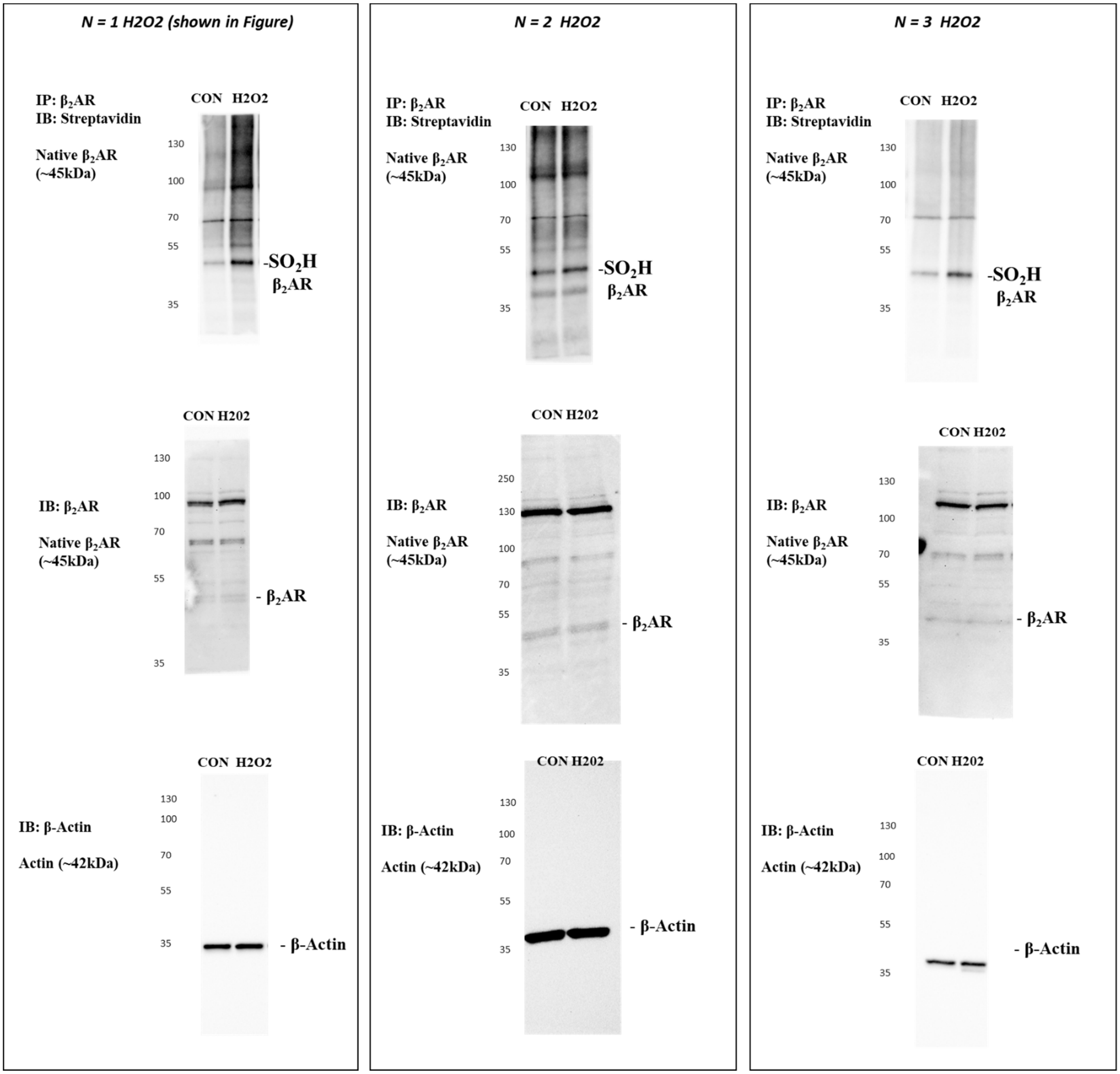

Full length immunoblot replicates from figure 2C:

A-SAEC – ISO blots

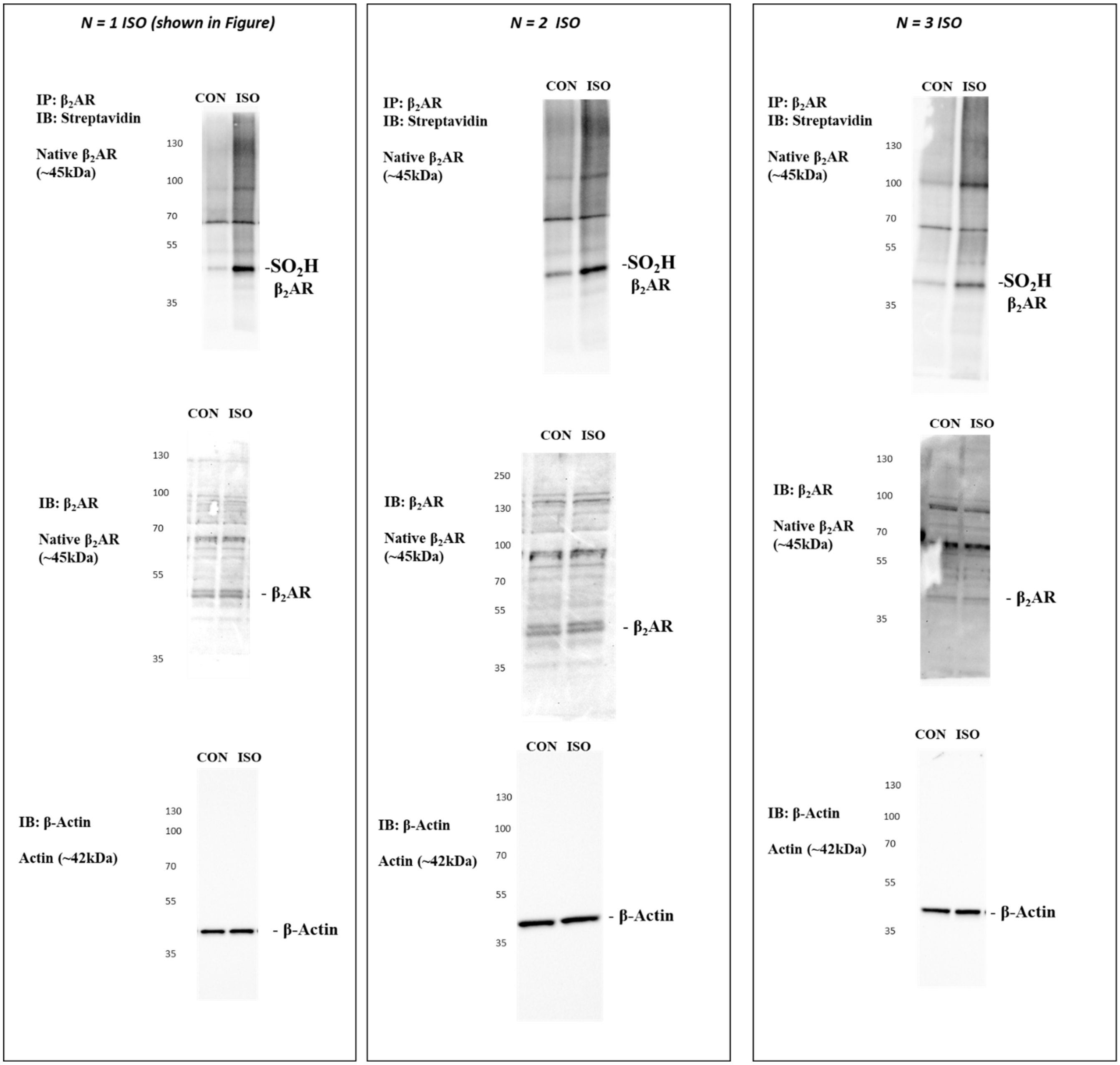

Full length immunoblot replicates from figure 2C:

A-SAEC – H_2O_2 blots

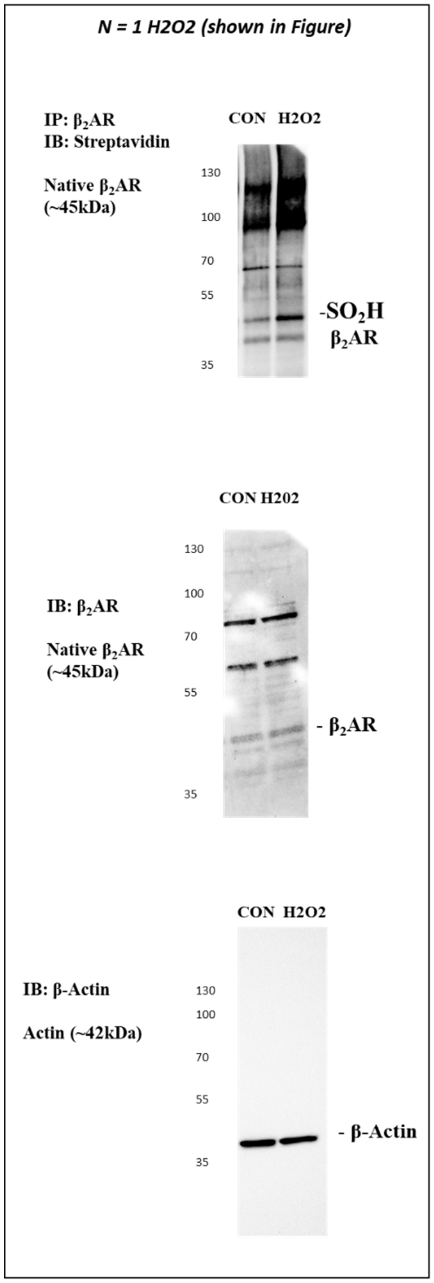

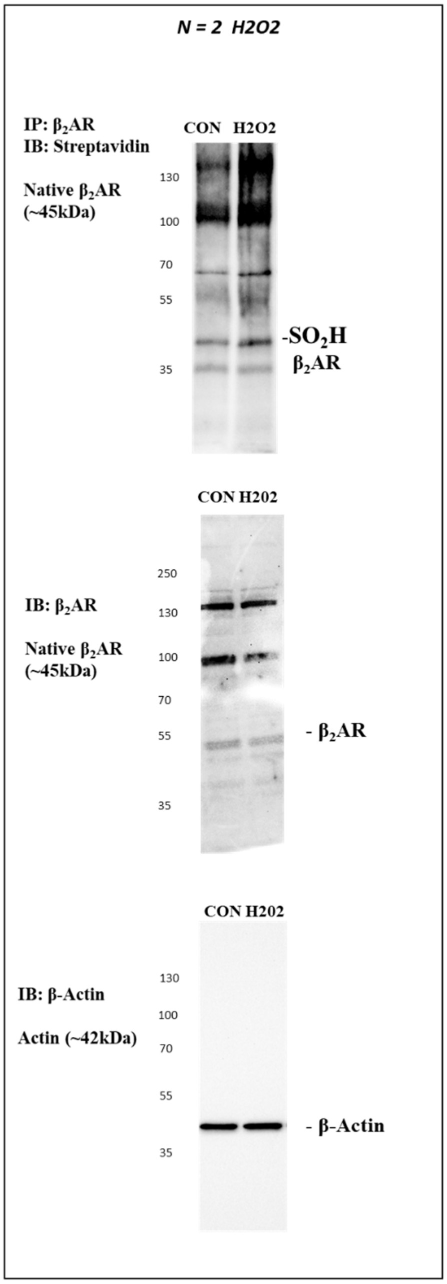

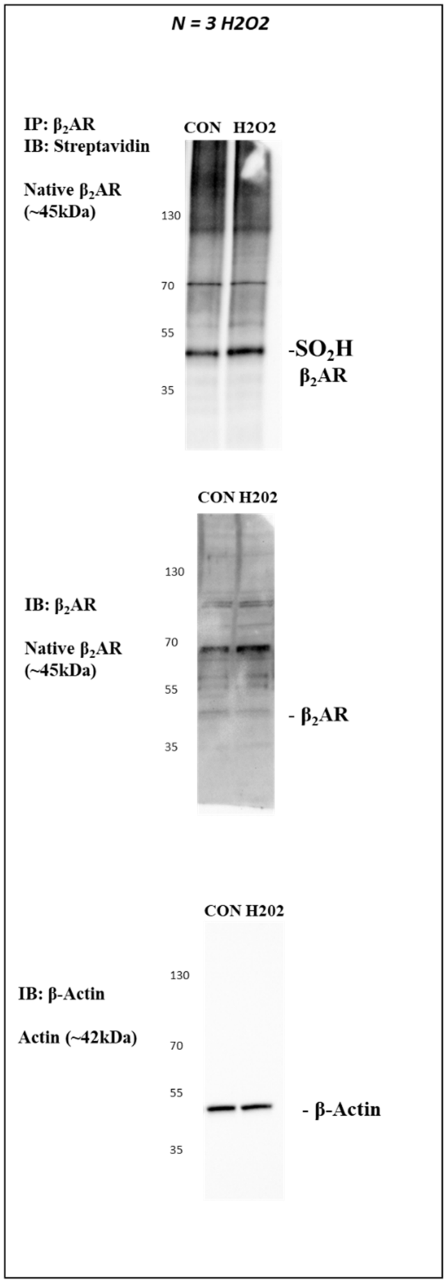

Full length immunoblot replicates from figure 6A:

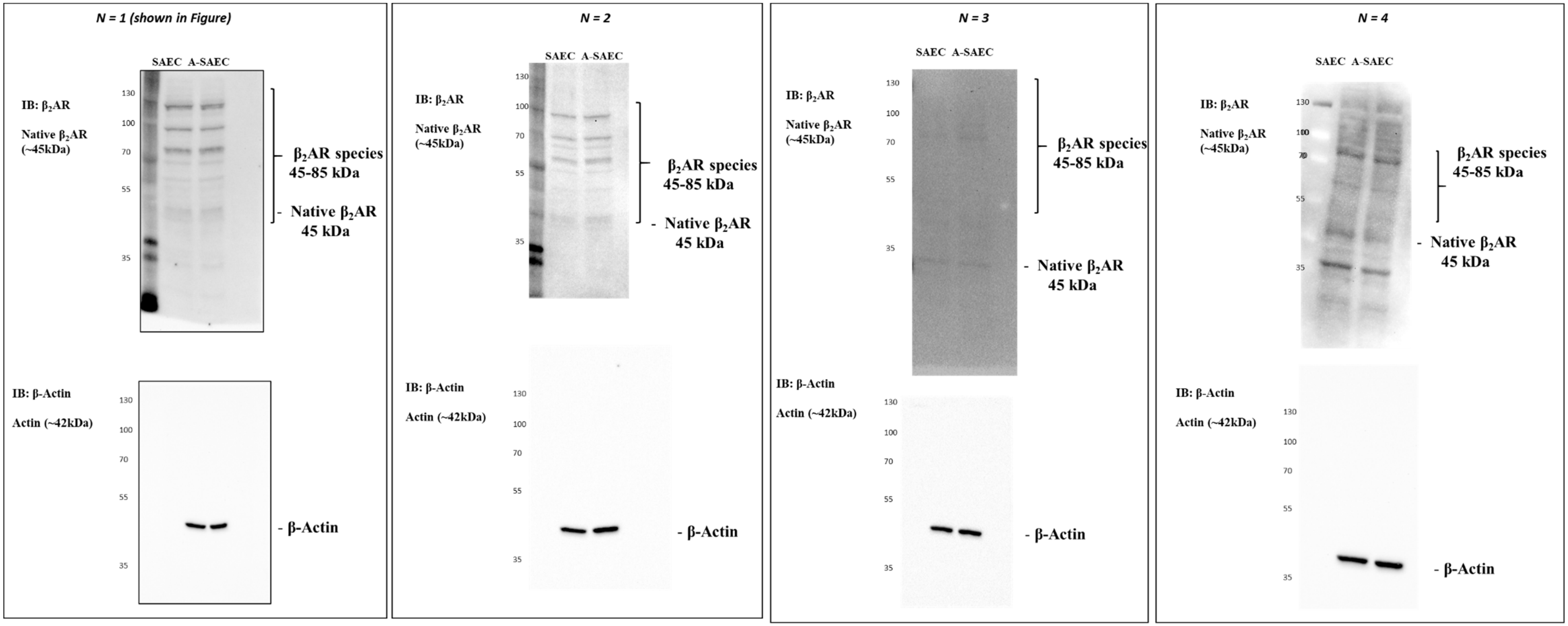

Full length immunoblot replicates from figure 6B:

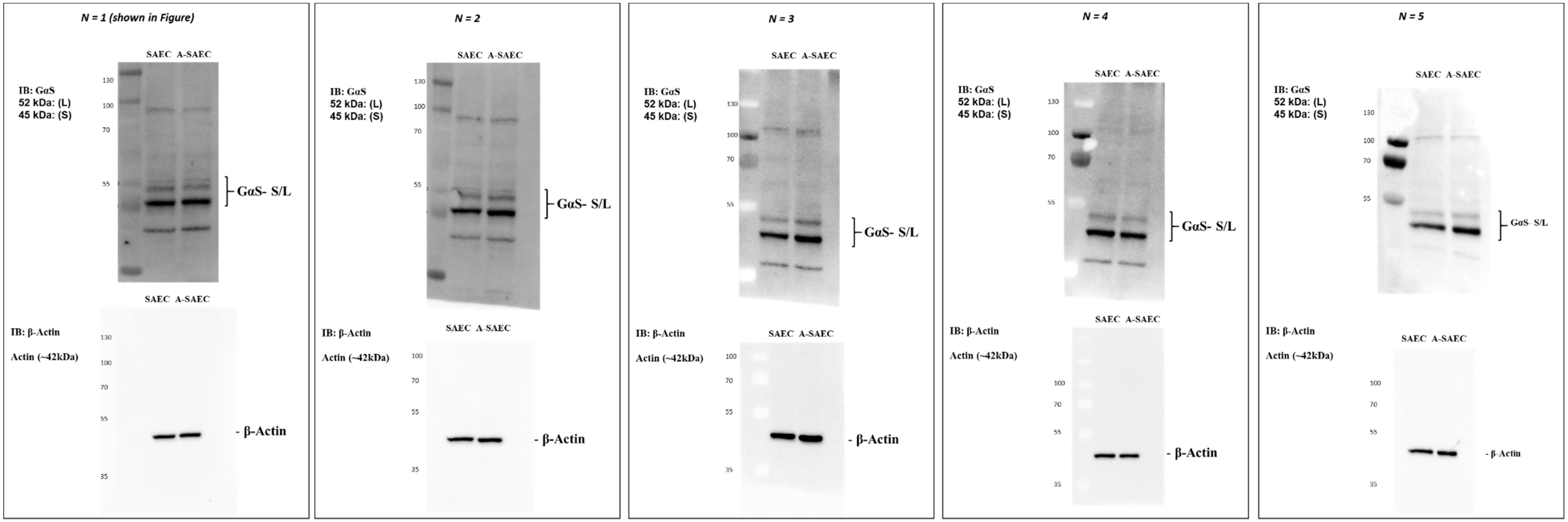

Full length immunoblot replicates from figure 6C:

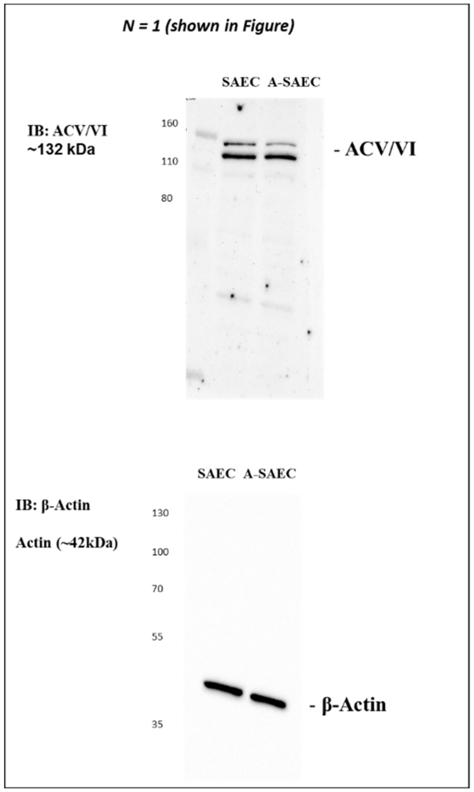

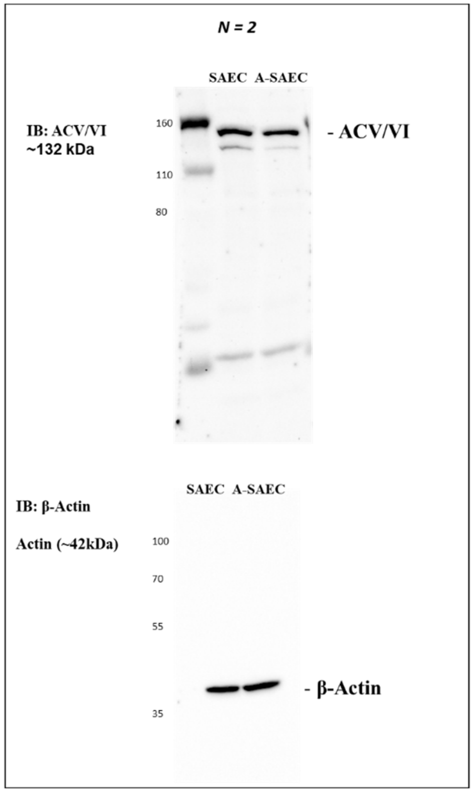

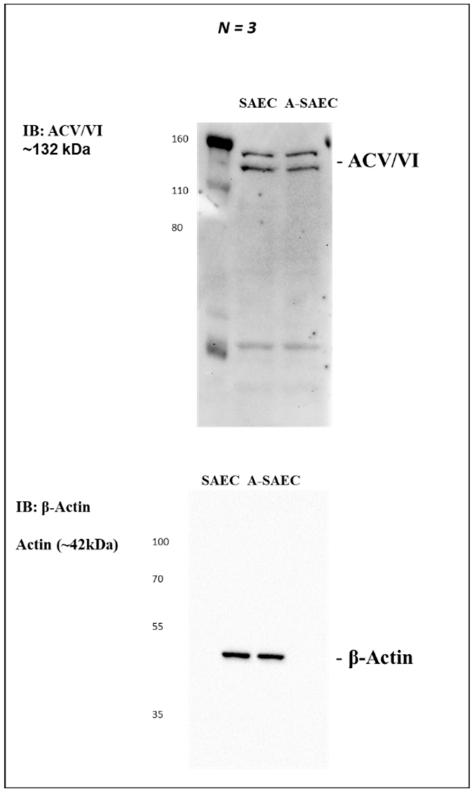

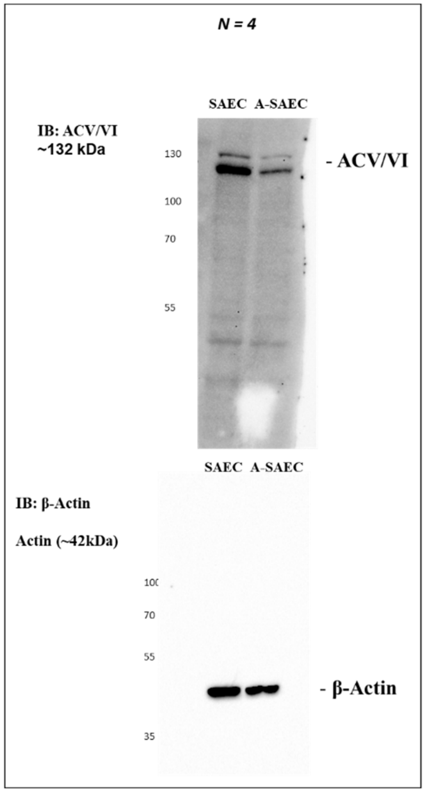

Full length immunoblot replicates from figure 6D:

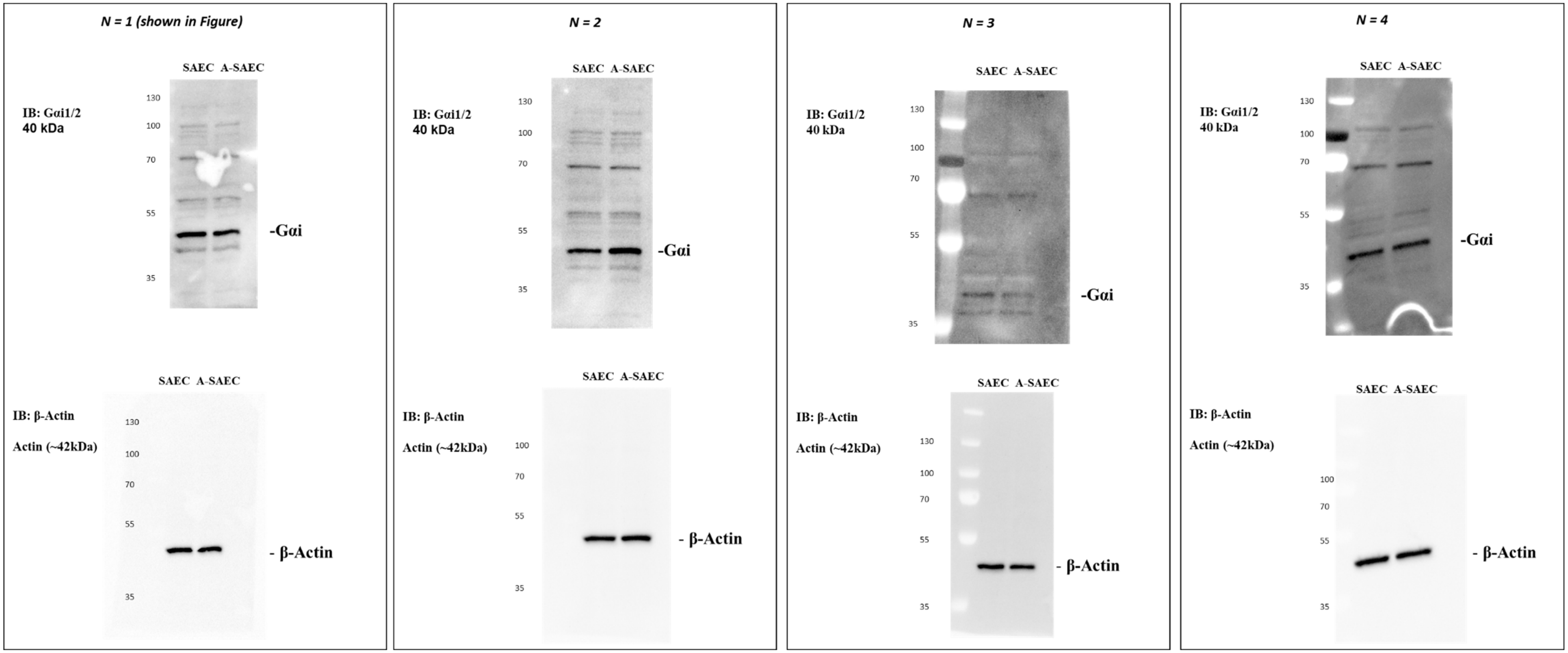

Full length immunoblot replicates from figure 6E:

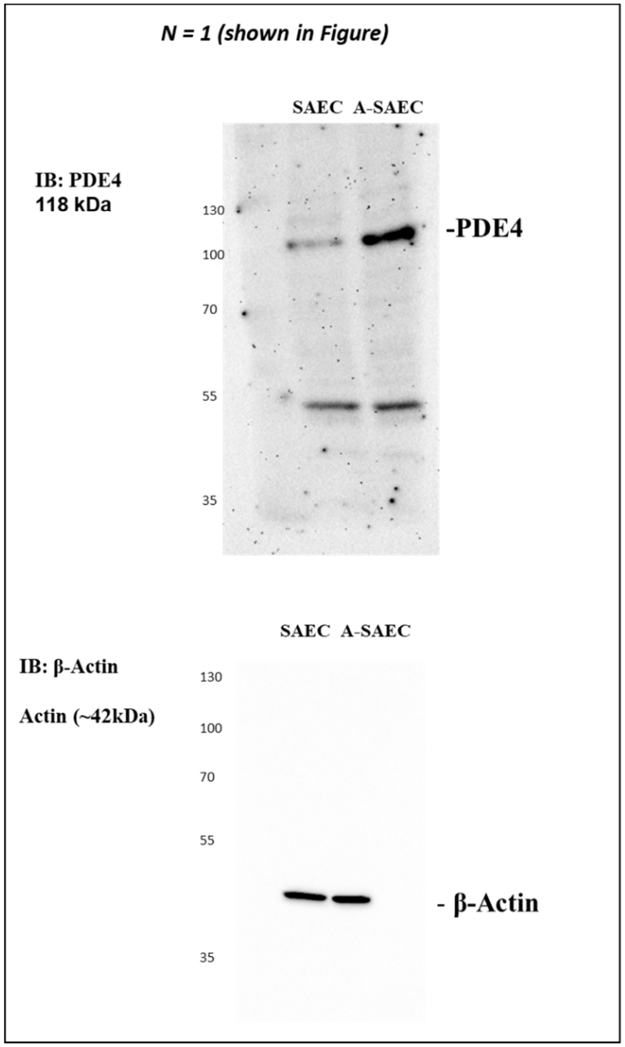

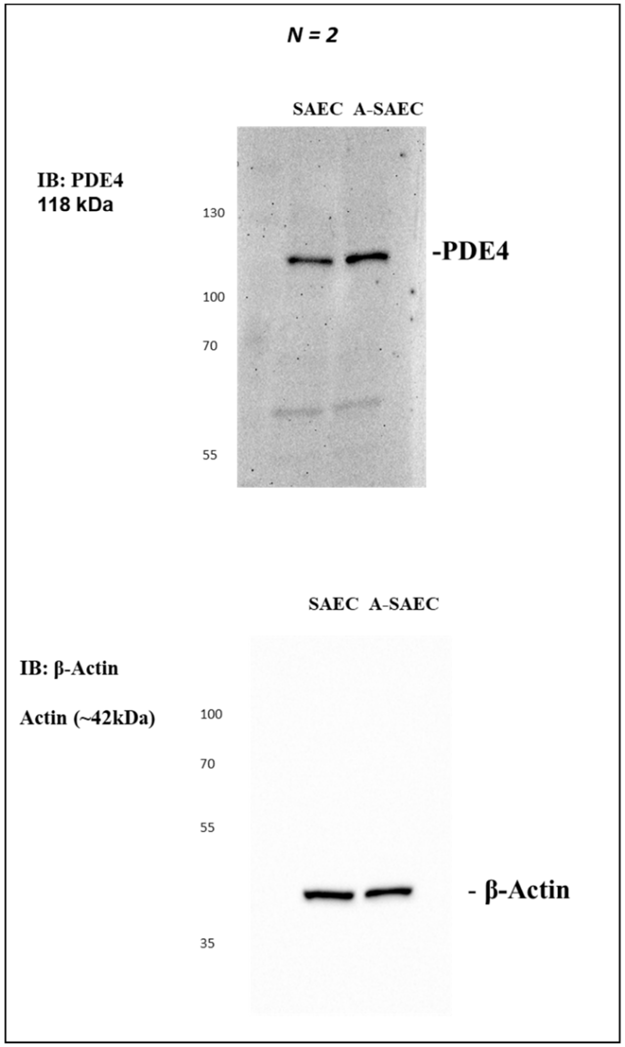

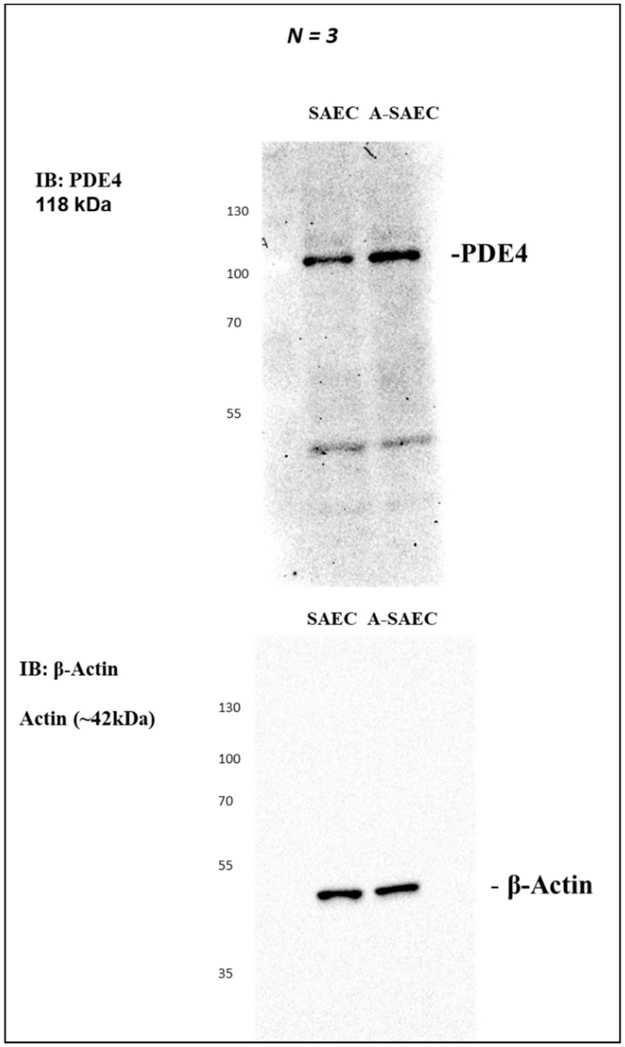

Full length immunoblot replicates from figure 6F (SOD1):

N = 1 (shown in Figure)

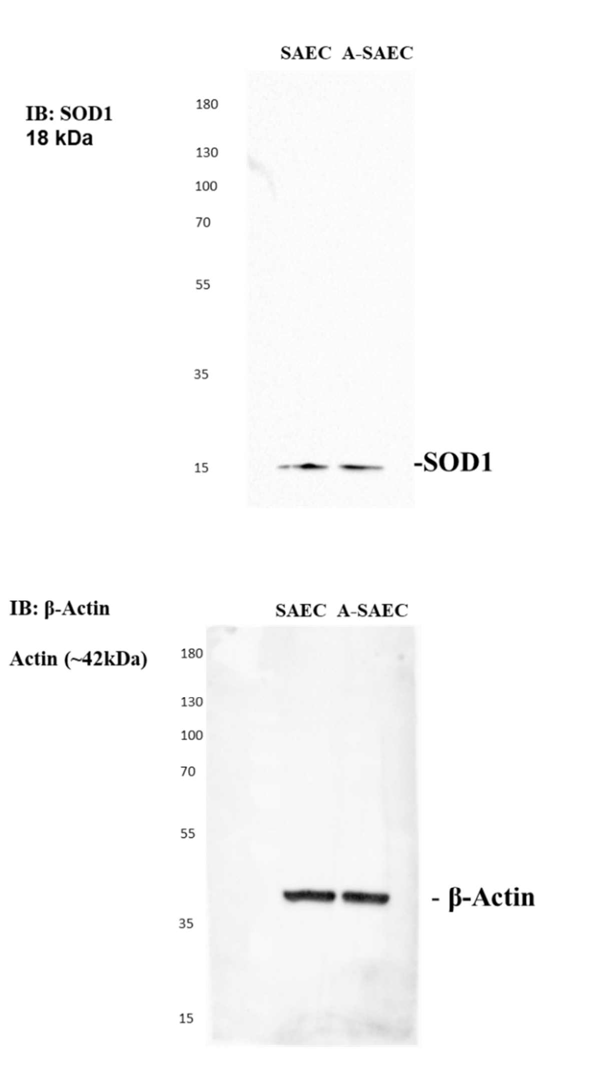

N = 2

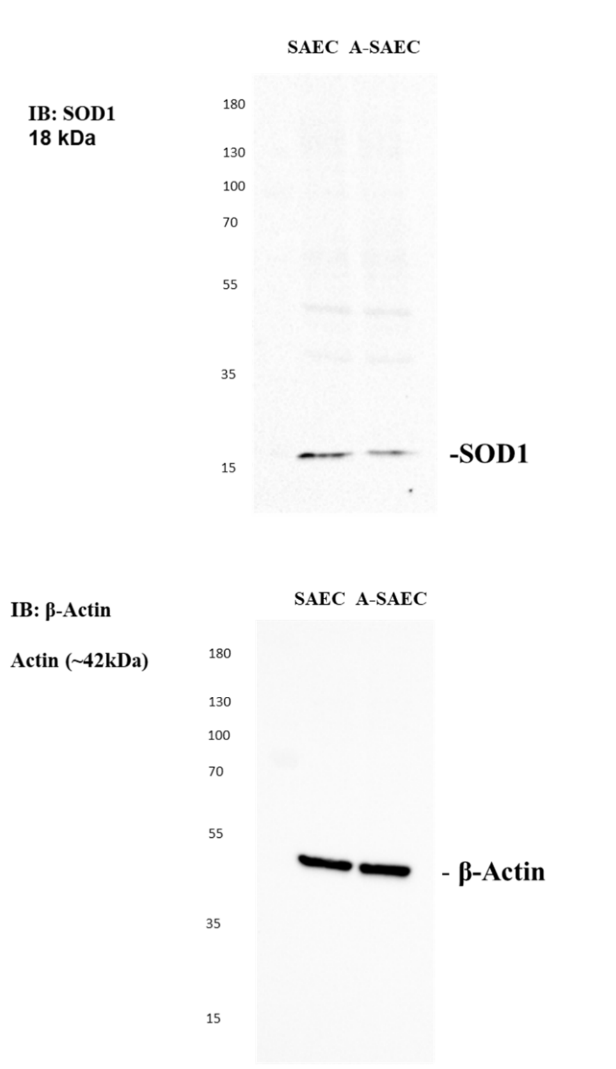

N = 3

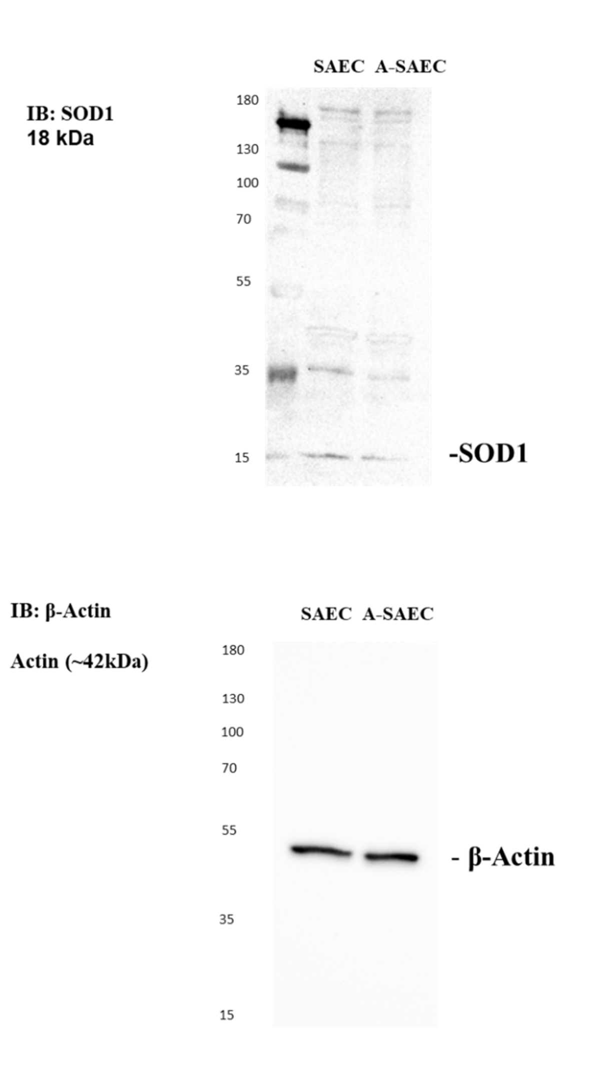

Full length immunoblot replicates from figure 6F (SOD2):

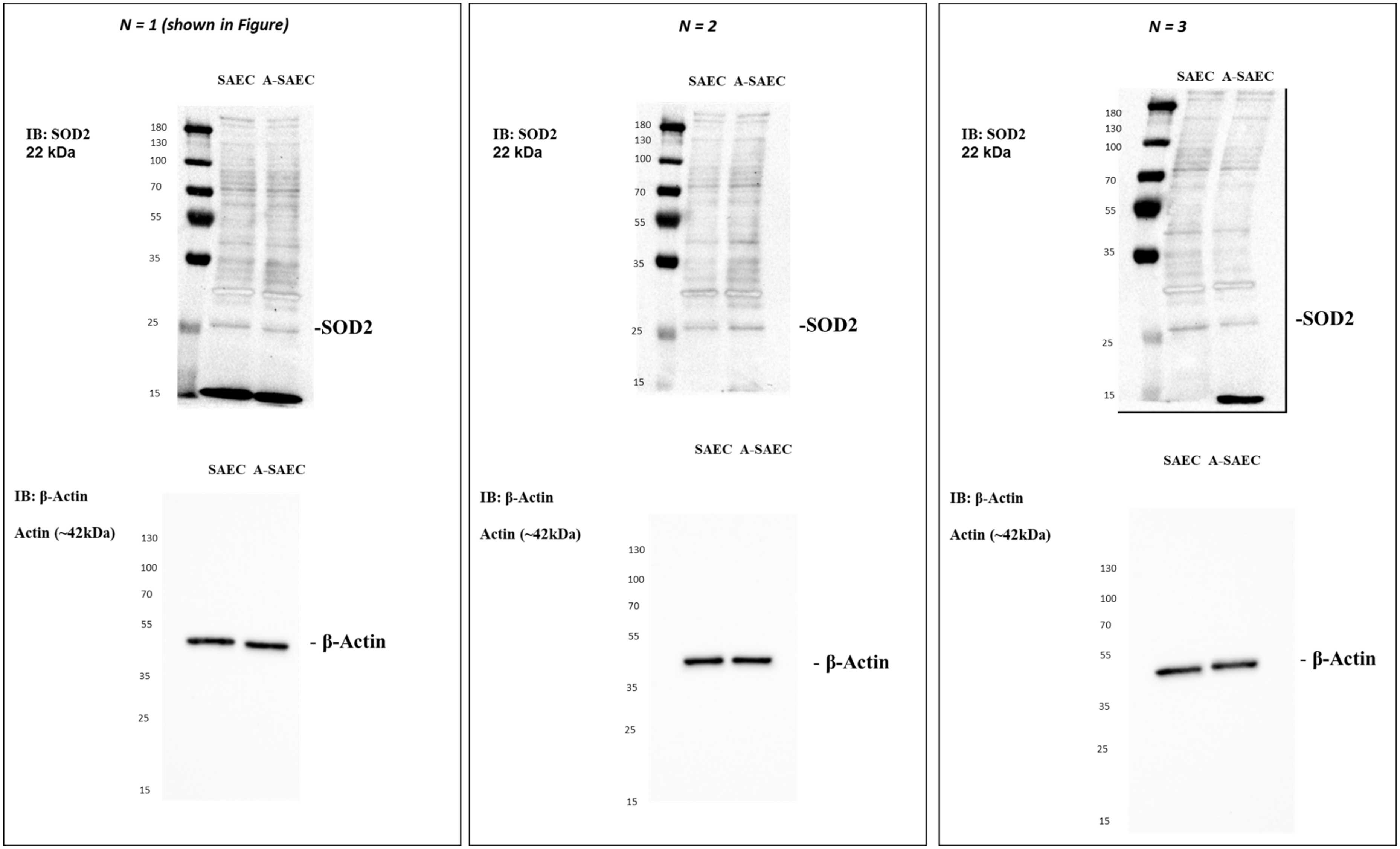

Full length immunoblot replicates from figure 6F (SOD3):

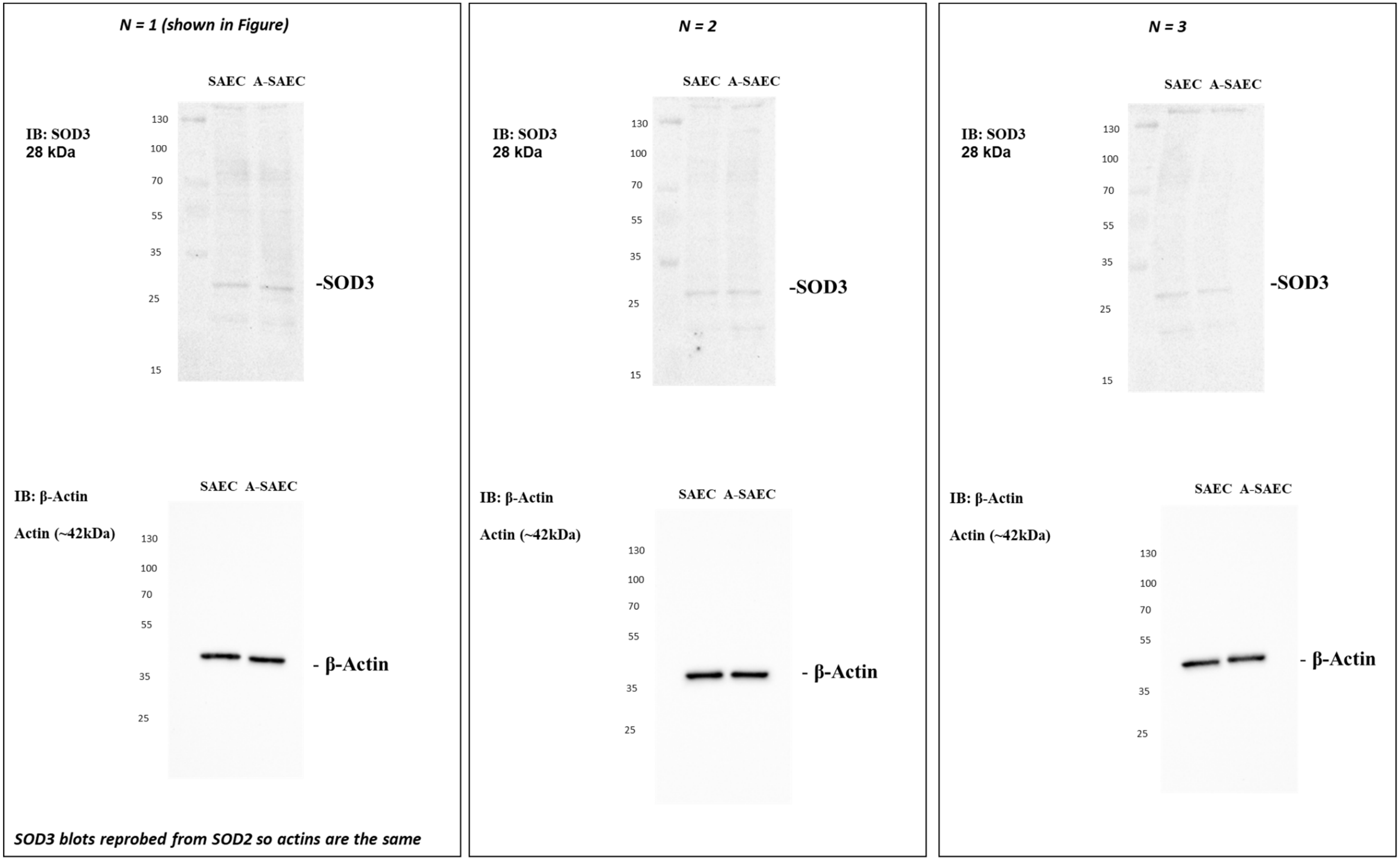

Full length immunoblot replicates from figure 6F (Catalase):

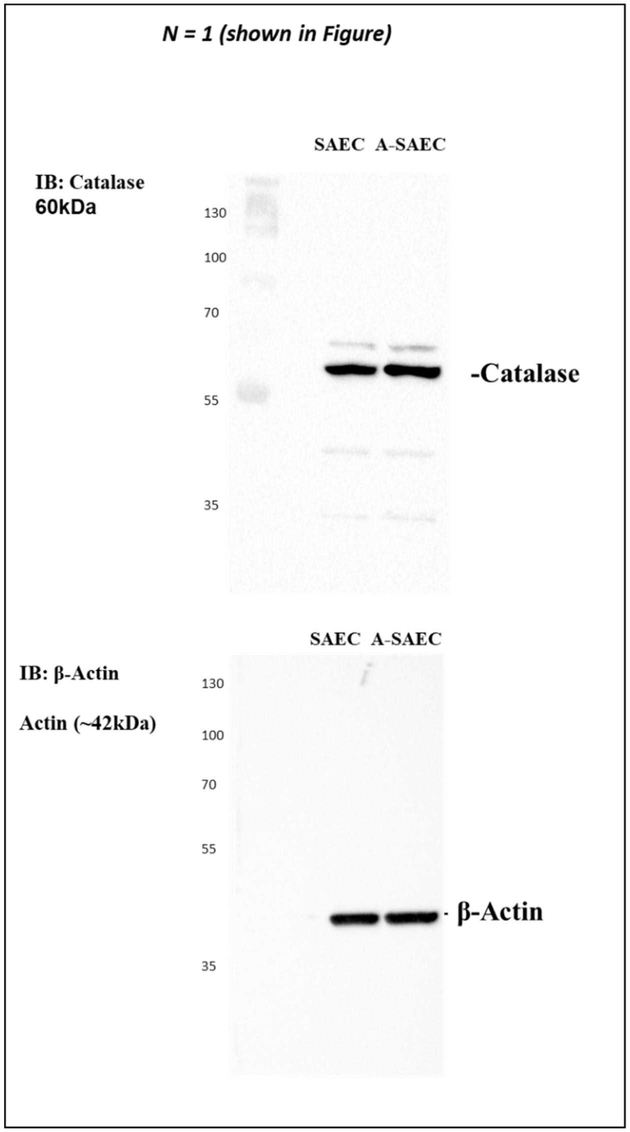

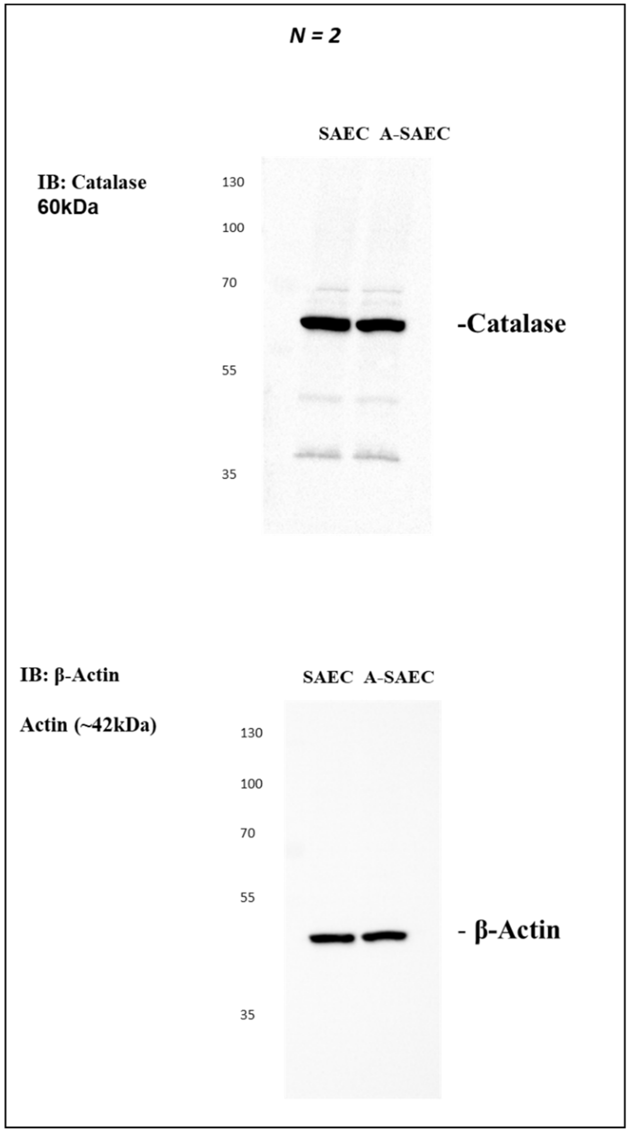

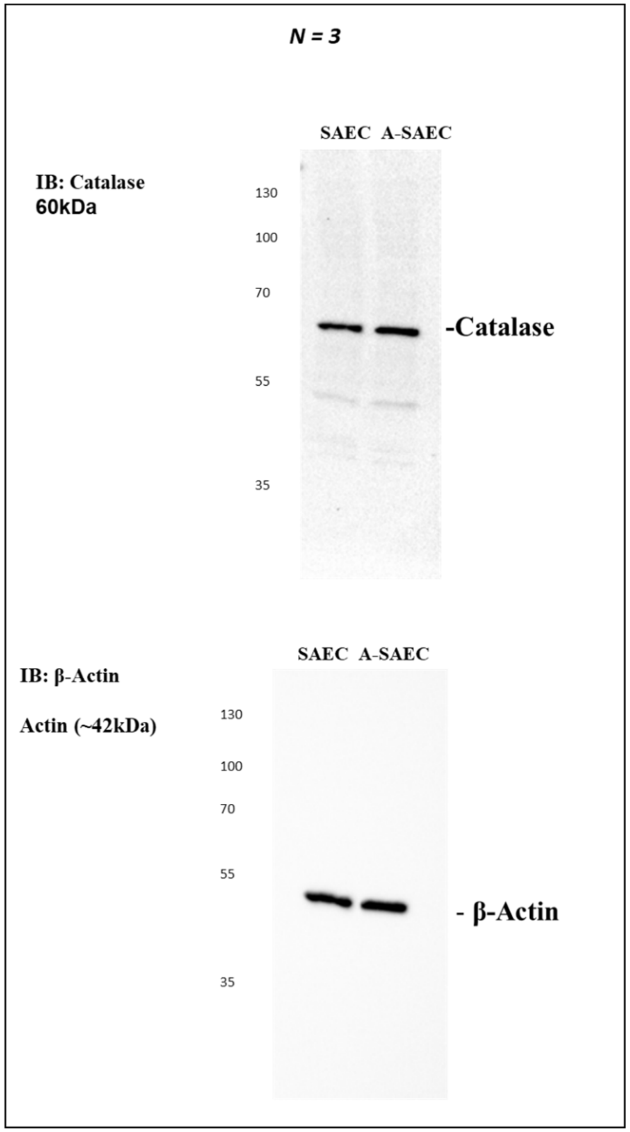

Supplement: Supplementary File [file NIHMS1940321-supplement-Supplementary_File.pdf]
